# Posterior minimally invasive scoliosis surgery versus the standard posterior approach for the management of adolescent idiopathic scoliosis: an updated meta-analysis

**DOI:** 10.1186/s13018-022-02954-4

**Published:** 2022-01-29

**Authors:** Honghao Yang, Xiangyuan Jia, Yong Hai

**Affiliations:** Department of Orthopedic Surgery, Beijing Chao-Yang Hospital, Capital Medical University, Gongti South Rd, No. 8, Beijing, 100020 China

**Keywords:** Minimally invasive scoliosis surgery, Posterior spinal fusion, Adolescent idiopathic scoliosis, Spinal deformity, Surgical management

## Abstract

**Background:**

Surgical management of adolescent idiopathic scoliosis (AIS) can be performed using standard posterior spinal fusion (PSF) or with a posterior minimally invasive approach. Minimally invasive scoliosis surgery (MISS) has several theoretical advantages, such as less tissue dissection, less blood loss, and earlier recovery. However, the difference in safety and effectiveness between MISS and PSF still needs to be clarified. This updated meta-analysis aimed to compare the outcomes of MISS and standard PSF for the management of AIS.

**Methods:**

A comprehensive literature search of PubMed, EMBASE, MEDLINE, and Cochrane Library without time restriction was performed to identify relevant studies. MISS and PSF were compared in terms of radiographic parameters, estimated blood loss (EBL), blood transfusion rate, operative time (ORT), length of hospital stay (LOS), overall Scoliosis Research Society-22 (SRS-22) score, postoperative pain, and complication rate.

**Results:**

A total of seven studies comprising 767 patients (329 MISS and 438 PSF) with AIS were included. MISS and PSF yielded comparable deformity correction at the last follow-up. There were no significant differences in the overall SRS-22 scores or complication rates between the groups. Nevertheless, greater restoration of thoracic kyphosis (WMD, 2.98; 95% CI 0.58 to 5.37, *P* = 0.015), less EBL (WMD, −218.76; 95% CI −256.41 to −181.11, *P* < 0.001), a lower blood transfusion rate (RR, 0.31; 95% CI 0.20 to 0.48, *P* < 0.001), a shorter LOS (WMD, −1.48; 95% CI −2.48 to −0.48, *P* = 0.004), less postoperative pain (WMD, 0.57; 95% CI 0.16 to 0.98, *P* = 0.006), and a longer ORT (WMD, 84.85; 95% CI 33.30 to 136.40, *P* = 0.001) were observed in the MISS group.

**Conclusion:**

Despite its inherent technical challenges, MISS is a feasible and effective alternative to standard PSF for AIS patients with moderate and flexible curves. MISS was associated with adequate deformity correction, better restoration of sagittal alignment, less EBL, fewer transfusions, shorter LOS, and better pain management compared to PSF. Further research is required to determine the detailed indications for the MISS procedure.

**Supplementary Information:**

The online version contains supplementary material available at 10.1186/s13018-022-02954-4.

## Background

Adolescent idiopathic scoliosis (AIS) is the most common spinal disorder affecting patients aged 10 years to maturity, and it has a high prevalence of 2% to 3% worldwide [1]. Management options include braces and specific exercises for mild scoliosis, while surgical intervention is needed for rapidly progressive cases [2]. The primary goals of spinal surgery are correcting deformity, restoring balance, and ultimately achieving solid arthrodesis [3]. Since the development of instrumentation, derotation maneuvers, and fusion techniques, open instrumented posterior spinal fusion (PSF) has been considered the standard procedure for the surgical treatment of AIS [4]. However, as a major surgery, PSF is associated with extensive blood loss, prolonged hospital stays, postoperative pain, and a risk of infection [5]. The long scar resulting from the incision is also a cosmetic problem for adolescent patients and may be accompanied by psychosocial distress [6]. Additionally, there are concerns regarding the paraspinal muscle morbidity caused by denervation [7]. Consequently, efforts have been made to limit the extent of surgical trauma.

Minimally invasive surgery (MIS) techniques have gained increasing popularity for the treatment of various traumatic or degenerative spinal disorders in the adult patient population [8, 9]. MIS has also been shown to be comparable to PSF for correcting adult spinal deformity and has the additional benefit of reduced tissue damage, less bleeding, and earlier mobilization and discharge [10]. Previous feasibility studies of MIS for deformity correction and their reported advantages have convinced surgeons to attempt to use posterior minimally invasive techniques for AIS [11–13]. Although Sarwahi et al. and de Bodman et al. successfully performed three-incision minimally invasive scoliosis surgery (MISS) for moderate AIS patients, inherent technical challenges are present in this procedure [11, 14]. AIS patients have much larger curves than patients with degenerative scoliosis, and more levels require instrumentation. The use of multiple stab incisions of the skin limits the access needed to perform adequate facetectomies and fusion [15]. Additionally, due to the significant vertebral rotation in AIS, it is difficult to identify the ideal pedicle screw trajectory without a sufficient view of anatomical landmarks. The resulting need for repeated fluoroscopy results in greater radiation exposure for surgeons and patients, which is especially harmful to children [16]. Moreover, the presence of a rigid curve, vertebral rotation, limited visualization, and soft tissue may make contoured rod passage and reduction maneuvers challenging [17].

A meta-analysis comparing the outcomes of MISS and standard PSF in the management of moderate AIS was conducted by Alhammoud et al. in 2019, and they concluded that there were no differences in curve correction, postoperative pain, or length of hospital stay (LOS) between the two procedures [18]. However, their conclusions may be limited due to the small number of subjects (only four studies with 42 patients in the MISS group and 65 patients in the PSF group were included). Since new comparative studies with more subjects have been published since that meta-analysis, we considered that an updated meta-analysis was necessary to provide valuable guidance regarding current surgical options for AIS [19–21].

This updated meta-analysis aimed to compare the radiographic outcomes, surgical information, clinical outcomes, and complications of MISS and standard PSF for the management of AIS.

## Methods

This study was designed according to the Preferred Reporting Items for Systematic Reviews and Meta-Analyses (PRISMA) guidelines [22]. This study is registered with PROSPERO (ID: CRD42020217823).

### Search strategy

The PubMed, EMBASE, MEDLINE, and Cochrane Library databases were searched using the following terms: (minimally invasive) AND (adolescent idiopathic scoliosis).

The literature search was last updated on April 26, 2021. Two reviewers (H.Y. and X.J.) independently screened titles, abstracts, and full texts. Any differences that arose were settled by discussion with a third party (Y.H.).

### Inclusion and exclusion criteria

The inclusion criteria in this meta-analysis were as follows: (1) target population: patients diagnosed with AIS; (2) intervention: MISS procedures were performed; (3) comparison: patients undergoing standard PSF procedures; and (4) outcomes: radiographic outcomes at the last follow-up, surgical information, clinical outcomes, and complications.

The exclusion criteria were as follows: (1) patients with a nonidiopathic etiology (e.g., neuromuscular or congenital scoliosis) and those undergoing revision spinal surgery; (2) patients undergoing thoracoscopic or laparoscopic surgery; (3) noncomparative studies; (4) reviews, case reports, and biomechanical studies; (5) duplicated publications; and (6) articles that were not published in English.

### Assessment of study quality

The quality of the studies was assessed independently by two reviewers (H.Y. and X.J.) using the Newcastle–Ottawa Scale (NOS), as recommended for retrospective studies by Cochrane Handbooks version 5.2.0 [23].

### Outcomes

Radiographic outcomes were the main curve Cobb angle and its correction rate, thoracic kyphosis (TK), lumbar lordosis (LL), coronal balance (CB), and sagittal vertical axis (SVA) at the last follow-up. Surgical information included estimated blood loss (EBL), blood transfusion rate, and operative time (ORT). The clinical outcomes were LOS, overall Scoliosis Research Society-22 (SRS-22) score, SRS-22 self-image/appearance score, and postoperative pain based on the SRS-22 pain score or the visual analog scale (VAS) score. Complications included the overall complication rate, surgical site infection, and hardware failure.

### Data extraction

Data extraction was performed by two reviewers independently (H.Y. and X.J.). Demographic and clinical information, including age, sex, curve types according to Lenke classification, and the number of fusion levels, was extracted; additionally, the sample size of each study was recorded [24]. This meta-analysis was performed for 17 variables. Continuous outcomes included the main curve Cobb angle and its correction rate, TK, LL, CB, SVA, EBL, ORT, LOS, overall SRS-22 score, SRS-22 self-image/appearance score, SRS-22 pain score, and VAS score. Because a greater SRS-22 pain score indicates less pain, while a greater VAS score indicates more severe pain, we used the negative value of VAS scores in the data analysis of postoperative pain [25, 26]. Dichotomous outcomes included the blood transfusion rate, overall complication rate, surgical site infection rate, and hardware failure rate. Outcomes reported by at least three studies were analyzed.

### Data analysis

All statistical analyses were performed using Stata 15.1. For continuous outcomes, the weighted mean difference (WMD) was utilized to estimate the effect. The effect measure of dichotomous outcomes was displayed as risk ratio (RR). Statistical heterogeneity among studies was evaluated using the I-square test and Cochran’s Q test. If the I^2^ value was less than 50% and the *P-*value was greater than 0.1, a fixed-effects model was performed. If the I^2^ value was greater than 50% or the *P-*value was less than 0.1, a sensitivity analysis was applied to assess the impact of each study. A Galbraith plot was used to visualize the heterogeneity [27]. If the I^2^ and the *P*-value could be reduced to less than 50% and 0.1, respectively, following the exclusion of one or two studies, these studies were omitted, and a fixed-effects model was used. Influence analysis was performed to investigate whether the exclusion of these studies would cause the effect size to be overestimated or underestimated [28]. If the heterogeneity could not be significantly diminished using these methods, the random-effects model was used. Subgroup analysis was performed to explore the potential sources of heterogeneity.

### Assessment of publication bias

Potential publication bias was assessed by the application of Egger’s test at the *P* < 0.10 level of significance [29]. If publication bias was indicated, we evaluated the number of missing studies in this meta-analysis using the trim-and-fill method and recalculated the pooled effect estimate with the addition of the missing hypothetical studies [30].

## Results

### Study selection

**Fig. 1:**
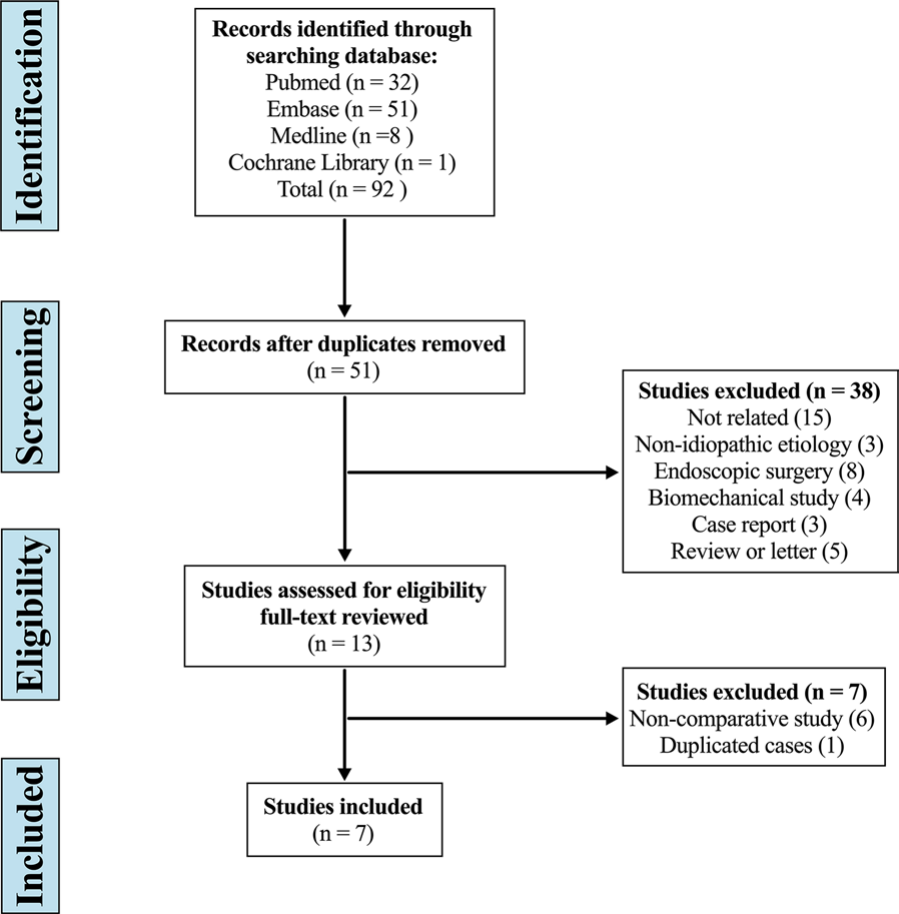
Flow diagram depicting the literature review, search strategy, and selection process

The systematic search yielded 92 studies, of which 41 were duplicates. Thirty-eight studies were excluded after screening of the title and abstract, and six studies were reasonably considered inappropriate after full-text review. Eventually, seven studies were finally included in this meta-analysis (Fig. 1) [15, 19–21, 31–33].

### Assessment of study quality

The quality of the included studies was assessed according to the NOS (Table 1). Of the seven studies, three were of high quality, with a score of 8, and four were of moderate quality, with a score of 7.

**Table 1:** Quality assessment of studies according to Newcastle–Ottawa Scale (NOS)

Author	Year	Selection	Comparability	Exposure	Total Score
Miyanji	2015	3	2	2	7
Sarwahi	2016	3	2	2	7
Zhu	2017	3	2	3	8
Urbanski	2019	3	2	2	7
Si	2020	3	2	3	8
Yang	2021	3	2	2	7
Sarwahi	2021	3	2	3	8

### Characteristics of the included studies

A total of seven studies comprising 767 patients with AIS were included. Of the patients, 329 underwent MISS procedures, and 438 underwent PSF procedures. The characteristics of the included studies are shown in Table 2. There were no significant differences in demographic data, number of fusion levels, main curve Cobb angle, TK, LL, CB, or SVA at baseline between the two groups.

**Table 2:** Basic characteristics of included studies

Study	Year	Design	Group	Sample size	Age (years)	Gender (M/F)	Lenke classification	Fusion Levels	Preoperative Cobb (°)	Preoperative TK (°)	Follow-up (months)
Miyanji	2015	Retrospective	MISS	23	16.8 ± 0.4	3/20	1 (20); 2 (2); 4 (1)	10.2	56.7 ± 1.6	20.5 ± 2.1	24.0
			PSF	23	16.4 ± 0.3	4/19	1 (12); 2 (8); 3 (3)	12.2	58.1 ± 1.6	22.6 ± 3.4	24.0
Sarwahi	2016	Retrospective	MISS	7	15.1 ± 3.2	1/6	1 (4); 2 (1); 5 (2)	10.0 ± 1.8	48.3 ± 8.3	20.7 ± 11.0	29.2 ± 2.3
			PSF	15	15.4 ± 2.1	2/13	1 (6); 2 (1); 4 (4); 5 (2); 6 (2)	12.3 ± 1.6	46.7 ± 3.3	24.0 ± 9.8	37.0 ± 7.4
Zhu	2017	Retrospective	MISS	15	16.5 ± 1.6	2/13	5 (15)	4.9 ± 0.5	48.3 ± 4.2	20.2 ± 6.1	27.1 ± 4.0
			PSF	30	15.1 ± 1.7	3/27	5 (30)	5.7 ± 0.5	50.9 ± 5.4	16.5 ± 6.8	32.9 ± 3.5
Urbanski	2019	Retrospective	MISS	4	15.5 ± 2.1	4/0	5 (4)	6.5 ± 0.9	57.3 ± 10.6	23.6 ± 7.6	NA
			PSF	4	21.3 ± 10.0	3/1	5 (4)	5.8 ± 0.4	47.0 ± 7.8	37.0 ± 16.1	NA
Si	2020	Retrospective	MISS	64	13.2 ± 1.7	20/44	1 (18); 2 (12); 3 (19); 4 (15)	8.4 ± 2.3	50.7 ± 8.8	29.2 ± 9.4	31.2 ± 4.7
			PSF	48	14.6 ± 1.9	14/34	1 (21); 2 (11); 3 (12); 4 (4)	6.2 ± 2.6	48.0 ± 8.4	28.7 ± 7.1	32.3 ± 6.3
Yang	2021	Retrospective	MISS	24	15.0 ± 1.9	0/24	1 (17); 3 (4); 5 (2); 6 (1)	12.3 ± 1.4	60.8 ± 9.4	35.0 ± 9.2	116.4 ± 0.0
			PSF	25	14.0 ± 1.5	0/25	1 (13); 2 (1); 3 (7); 4 (1); 5 (1); 6 (2)	12.1 ± 1.5	62.1 ± 12.9	26.5 ± 12.1	55.2 ± 2.4
Sarwahi	2021	Retrospective	MISS	192	15.0 ± 1.9	25/167	NA	11.3 ± 2.2	55.7 ± 9.0	24.7 ± 12.7	> 24.0
			PSF	293	15.0 ± 2.4	66/227	NA	11.7 ± 2.2	53.3 ± 11.2	25.3 ± 12.7	> 24.0

*TK* thoracic kyphosis, *MISS* minimally invasive scoliosis surgery, *PSF* posterior spinal fusion, *NA* not available

### Surgical technique

As reported in the included studies, PSF was performed using a standard posterior approach with subperiosteal muscle dissection, facetectomy/decortication, pedicle screw insertion, rod placement, derotation, and correction maneuvers. MISS was performed using one to three small (3–5 cm) midline skin incisions. Facet joints were exposed using the Wiltse approach. Three to four segments (6–8 pedicle screws) were instrumented per skin incision, followed by subfascial rod placement and correction maneuvers with posterolateral fusion around the facets.

## Radiographic outcomes

### Main curve Cobb angle

**Fig. 2:**
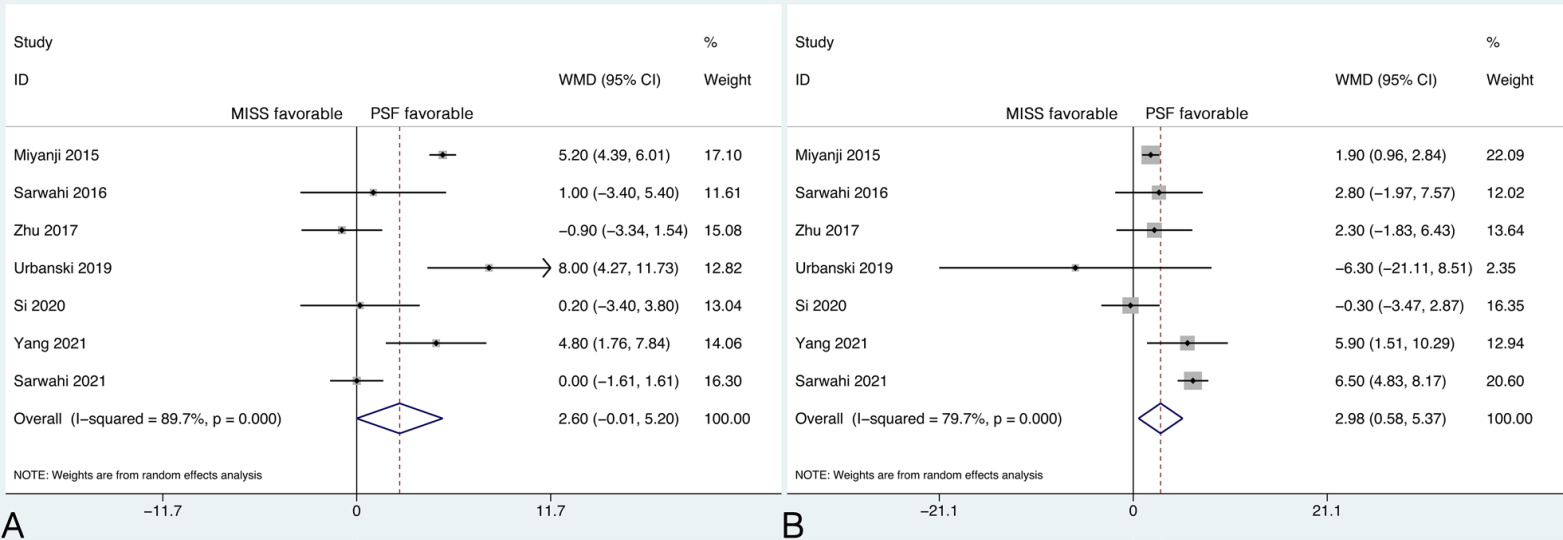
**A** Forest plot of the main curve Cobb angle at the last follow-up; **B** Forest plot of the thoracic kyphosis at the last follow-up

Seven studies reported the main curve Cobb angle at the last follow-up, and significant heterogeneity was detected (*P* < 0.001, *I*^2^ = 89.7%). However, further sensitivity analysis did not identify a study that could significantly affect the heterogeneity. Therefore, the random-effects model was applied. The pooled results revealed no significant difference between groups in the main curve Cobb angle at the last follow-up (WMD, 2.60; 95% CI −0.01 to 5.20, *P* = 0.051) (Fig. 2A).

### Correction rate of main curve Cobb angle

Seven studies reported the correction rate of the main curve Cobb angle, and significant heterogeneity was detected (*P* < 0.001, *I*^2^ = 92.8%). Therefore, a further sensitivity analysis was performed. The Galbraith plot indicated that the source of heterogeneity was the studies by Miyaji et al. [31] and by Urbanski et al. [33] (Additional file 1). Miyaji et al. [31] emphasized that the correction rate of MISS in their study may have been impacted by the learning curve when applying this new technique, while the difference in the preoperative main curve Cobb angle between the MISS and PSF groups was 10.3° in the study by Urbanski et al. [33], which was significantly greater than that of other studies (range 1.3°-2.7°) and may have skewed the correction rate. When the studies by Miyanji et al. [31] and by Urbanski et al. [33] were omitted from the meta-analysis, the heterogeneity was not significant (*P* = 0.164, *I*^2^ = 38.7%). An influence analysis indicated that the exclusion of the study by Miyaji et al. [31] would significantly affect the effect size (Additional file 2).

The remaining five studies included 302 patients in the MISS group and 411 patients in the PSF group. The pooled results revealed no significant difference in the correction rate between groups (WMD, −0.01; 95% CI −0.03 to 0.01, *P* = 0.518).

### Thoracic kyphosis

Seven studies reported TK at the last follow-up, and significant heterogeneity was detected (*P* < 0.001, *I*^2^ = 79.7%). However, further sensitivity analysis did not identify a study that could significantly affect the heterogeneity. Therefore, the random-effects model was applied. The mean TK at the last follow-up was 25.80° (95% CI 21.82 to 29.77) in the MISS group and 22.71° (95% CI 20.77 to 24.66) in the PSF group. The pooled results revealed that the TK at the last follow-up was significantly greater in the MISS group than in the PSF group (WMD, 2.98; 95% CI 0.58 to 5.37, *P* = 0.015) (Fig. 2B).

### Lumbar lordosis, coronal balance, and sagittal vertical axis

The pooled results revealed that there were no significant differences between groups in LL (WMD, -2.18; 95% CI -6.08 to 1.71, *P* = 0.272), CB (WMD, −0.56; 95% CI −1.41 to 0.30, *P* = 0.201), or SVA (WMD, 1.13; 95% CI −0.54 to 2.80, *P* = 0.186) at the last follow-up.

## Surgical information

### Estimated blood loss

Seven studies reported EBL, and significant heterogeneity was detected (*P* = 0.003, *I*^2^ = 69.5%). Therefore, further sensitivity analysis was performed. The Galbraith plot indicated that the source of heterogeneity was the study by Yang et al. [20] (Additional file 3). The difference in EBL between the MISS and PSF groups was 1224.0 mL in the study by Yang et al. [20], which was significantly greater than that in other studies (range 166.7–333.3 mL). Additionally, Yang et al. [20] reported that the mean EBL in the PSF group was 2503.0 mL, which was significantly higher than that reported in the other included studies (range 418.0–883.3 mL) and in a previous study (907 ± 775 mL) [34]. When the study by Yang et al. [20] was omitted from the meta-analysis, the heterogeneity was not significant (*P* = 0.111, *I*^2^ = 44.2%). Influence analysis indicated that the exclusion of this study would not cause the effect size to be overestimated or underestimated (Additional file 4).

When the fixed-effects model was used, the weight of the study by Miyanji et al. [31] was too large (81.82%), while the sample size was relatively small (23 MISS vs. 23 PSF), which may cause the effect size to be underestimated. To put greater weight on the studies with larger sample sizes, a random-effects model was used.

**Fig. 3:**
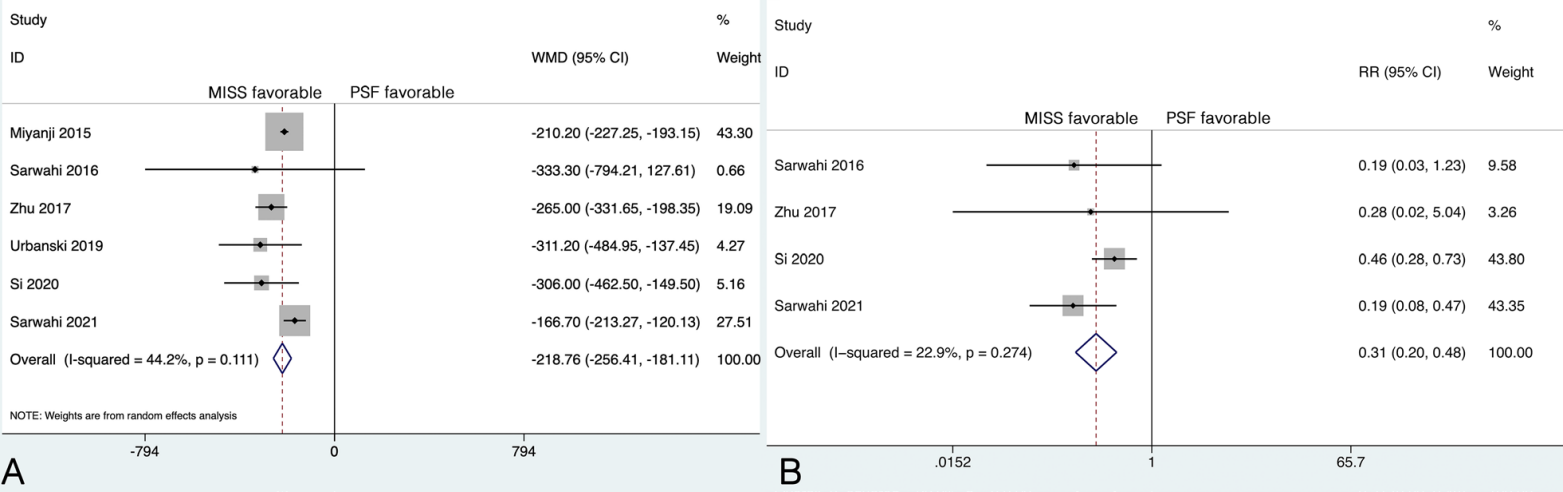
**A** Forest plot of the estimated blood loss; **B** Forest plot of the blood transfusion rate

The remaining six studies included 305 patients in the MISS group and 413 patients in the PSF group. The mean EBL was 288.25 mL (95% CI 204.96 to 371.54) in the MISS group and 517.19 mL (95% CI 456.44 to 577.94) in the PSF group. The pooled results revealed that the EBL in the MISS group was significantly less than that in the PSF group (WMD, −218.76; 95% CI −256.41 to −181.11, *P* < 0.001) (Fig. 3A). Regardless of the model that was used, the statistical significance did not change.

### Blood transfusion rate

Four studies reported the blood transfusion rate. There were 278 patients in the MISS group and 386 patients in the PSF group, with no substantial heterogeneity between groups (*P* = 0.274, *I*^2^ = 22.9%). The mean blood transfusion rate was 8.0% (95% CI 0.0% to 27.0%) in the MISS group and 35.0% (95% CI 9.0% to 66.0%) in the PSF group. The pooled results revealed a significantly lower blood transfusion rate in the MISS group than in the PSF group (RR, 0.31; 95% CI 0.20 to 0.48, *P* < 0.001) (Fig. 3B).

### Operative time

**Fig. 4:**
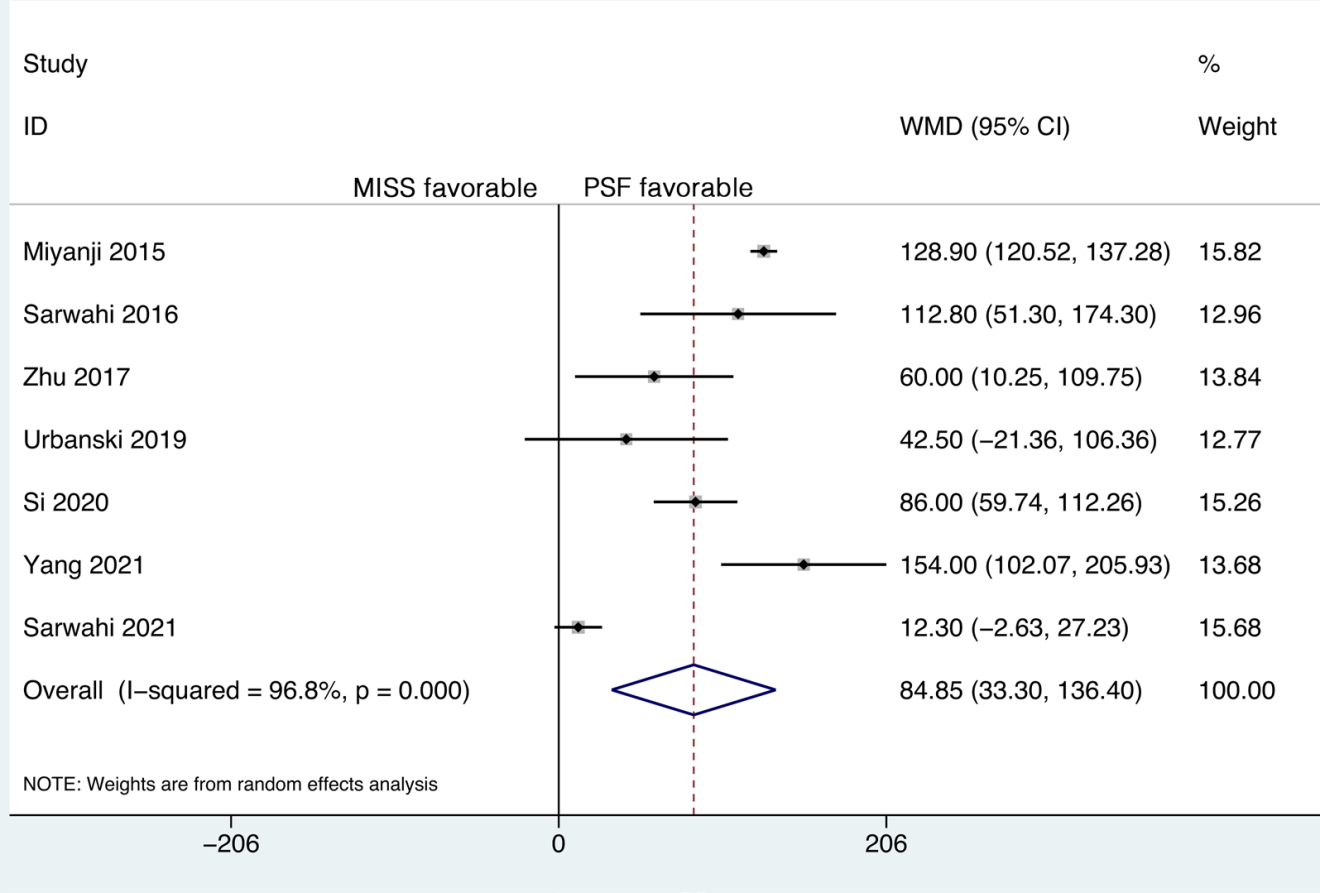
Forest plot of the operative time

Seven studies reported the ORT, and significant heterogeneity was detected (*P* < 0.001, *I*^2^ = 96.8%). However, further sensitivity analysis did not identify a study that could significantly affect the heterogeneity. Therefore, the random-effects model was applied. The mean ORT was 376.40 min (95% CI 293.35 to 459.45) in the MISS group and 287.60 min (95% CI 237.42 to 337.79) in the PSF group. The pooled results revealed a significantly longer ORT in the MISS group than in the PSF group (WMD, 84.85; 95% CI 33.30 to 136.40, *P* = 0.001) (Fig. 4).

## Clinical outcomes

### Length of hospital stay

Five studies reported the LOS, and significant heterogeneity was detected (*P* < 0.001, *I*^2^ = 94.9%). However, further sensitivity analysis did not identify a study that could significantly affect the heterogeneity. Therefore, the random-effects model was applied.

**Fig. 5:**
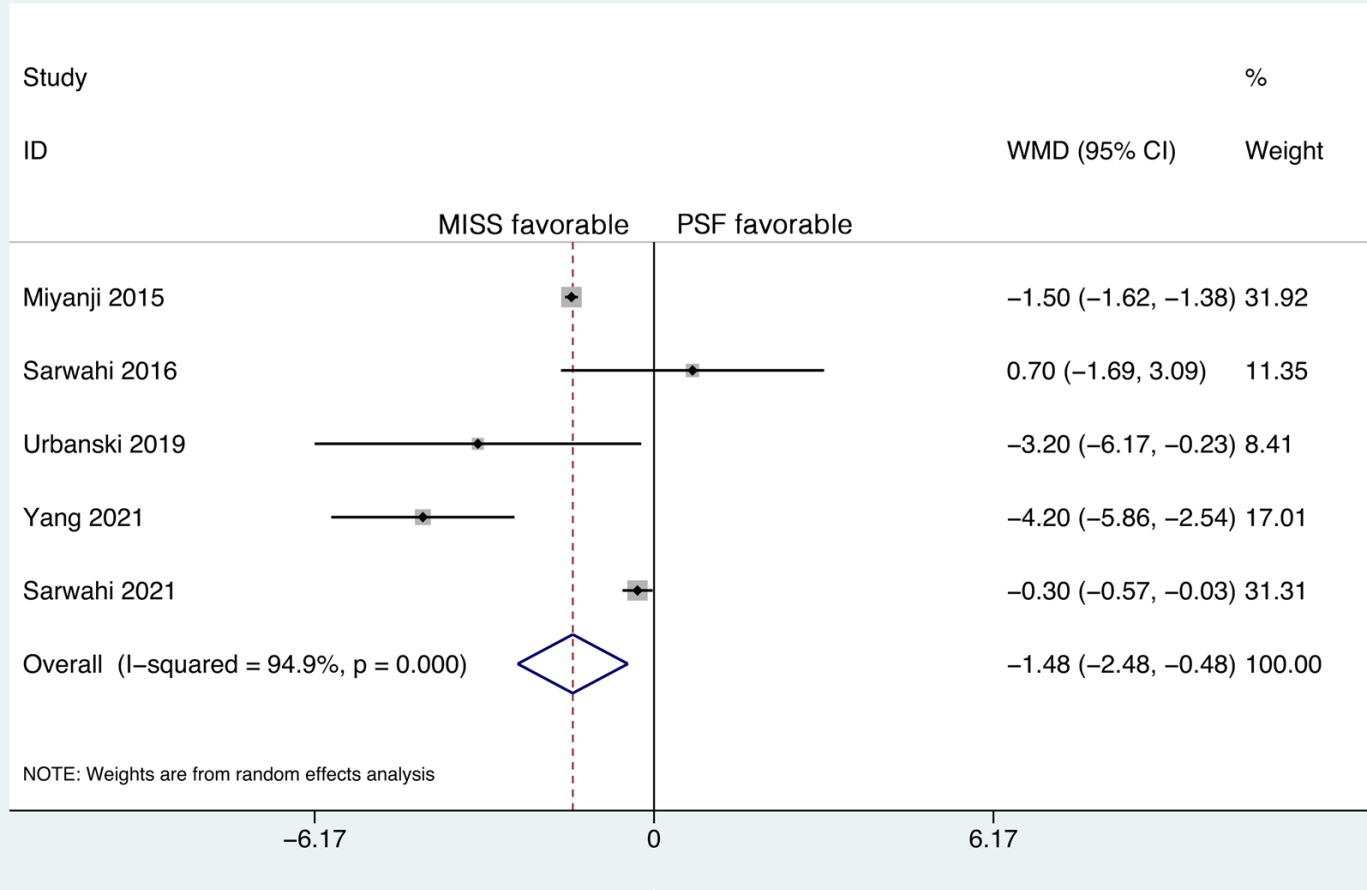
Forest plot of the length of hospital stay

The five studies included 250 patients in the MISS group and 351 patients in the PSF group. The mean LOS was 6.11 days (95% CI 4.80 to 7.41) in the MISS group and 7.69 days (95% CI 6.29 to 9.10) in the PSF group. The pooled results revealed a significantly shorter LOS in the MISS group than in the PSF group (WMD, -1.48; 95% CI -2.48 to -0.48, *P* = 0.004) (Fig. 5).

### SRS-22 score

Four studies reported the overall SRS-22 score after surgery, and significant heterogeneity was detected (*P* < 0.001, *I*^2^ = 90.8%). However, further sensitivity analysis did not identify a study that could significantly affect the heterogeneity. Therefore, the random-effects model was applied. The four studies included 126 patients in the MISS group and 126 patients in the PSF group. The mean overall SRS-22 score was 4.21 (95% CI 4.18 to 4.25) in the MISS group and 4.11 (95% CI 3.90 to 4.32) in the PSF group. The pooled results revealed no significant difference in the overall SRS-22 score between groups (WMD, 0.13; 95% CI −0.14 to 0.41, *P* = 0.339).

Only three of the four studies reported the SRS-22 self-image/appearance score and the SRS-22 pain score, including 103 patients in the MISS group and 103 patients in the PSF group. The pooled results revealed no significant difference in the SRS-22 self-image/appearance score between groups (WMD, 0.13; 95% CI -0.29 to 0.54, *P* = 0.546).

### Postoperative pain

Five studies reported postoperative pain. The studies included 146 patients in the MISS group and 168 patients in the PSF group. The SRS-22 pain score was utilized by three studies, and the VAS score was utilized by two studies. Significant heterogeneity was detected (*P* = 0.001, *I*^2^ = 77.3%), and a random-effects model was applied. The pooled results revealed significantly less postoperative pain in the MISS group than in the PSF group (WMD, 0.57; 95% CI 0.16 to 0.98, *P* = 0.006).

**Fig. 6:**
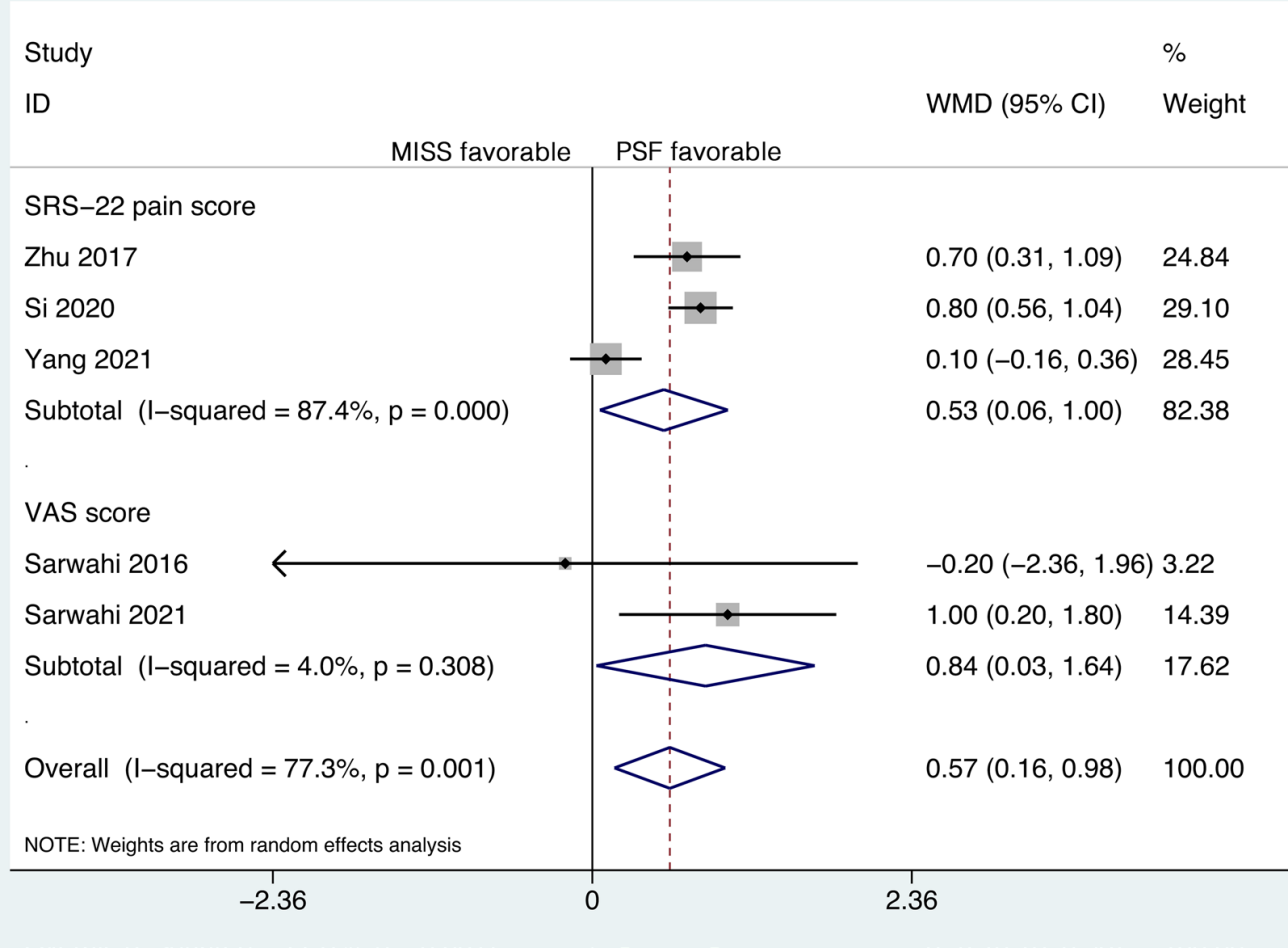
Forest plot of the postoperative pain

To eliminate the influence of different tools, a subgroup analysis was performed. The pooled results revealed significantly less postoperative pain in the MISS group than in the PSF group according to both the SRS-22 pain score (WMD, 0.53; 95% CI 0.06 to 1.00, *P* = 0.028) and the VAS score (WMD, 0.84; 95% CI 0.03 to 1.64, *P* = 0.042) (Fig. 6).

## Complications

### Overall complication rate

Complications, including surgical site infection, hardware failure, wound dehiscence, pseudarthrosis, and hemothorax, were reported by six studies that included 325 patients in the MISS group and 434 patients in the PSF group. No substantial heterogeneity was detected (*P* = 0.496, *I*^2^ = 0.0%). The pooled results revealed no significant difference in the overall complication rate between groups (RR, 1.13; 95% CI 0.77 to 1.67, *P* = 0.521).

### Surgical site infection

Surgical site infection, including superficial and deep wound infection, was reported by six studies with 325 patients in the MISS group and 434 patients in the PSF group. No substantial heterogeneity was detected (*P* = 0.563, *I*^2^ = 0.0%). The pooled results revealed no significant difference in the surgical site infection rate between groups (RR, 1.19; 95% CI 0.59 to 2.40, *P* = 0.633).

### Hardware failure

Hardware failure, including screw dislodgement, rod dislodgement, and rod breakage, was reported by five studies with 310 patients in the MISS group and 404 patients in the PSF group. No substantial heterogeneity was detected (*P* = 0.679, *I*^2^ = 0.0%). The pooled results revealed no significant difference in the hardware failure rate between groups (RR, 1.12; 95% CI 0.40 to 3.19, *P* = 0.828).

### Subgroup analysis by curve type

To explore whether the source of heterogeneity was the variety of curve types in AIS patients, we performed a subgroup analysis of the main curve Cobb angle, correction rate, TK, EBL, and ORT according to the curve type (Additional file 5). The heterogeneity of TK and EBL was significantly decreased after the subgroup analysis. The TK at the last follow-up was significantly greater in the MISS group (WMD, 6.03; 95% CI 4.46 to 7.60, *P* < 0.001) for patients with mixed curve types (Lenke types 1–6). EBL was significantly lower in the MISS group for Lenke types 1–4 (WMD, -225.22; 95% CI −293.48 to −156.95, *P* < 0.001) and Lenke type 5 patients (WMD, −270.93; 95% CI −333.16 to −208.70, *P* < 0.001). The ORT was significantly longer in the MISS group for Lenke types 1–4 (WMD, 109.33; 95% CI 67.45 to 151.21, *P* < 0.001) and Lenke type 5 patients (WMD, 53.39; 95% CI 14.14 to 92.64, *P* = 0.008), while there was no significant difference for patients with mixed curve types (Lenke types 1–6).

### Subgroup analysis by fusion levels

To explore whether the source of heterogeneity was the different fusion levels in AIS patients, we performed a subgroup analysis of the main curve Cobb angle, correction rate, TK, EBL, and ORT according to the fusion levels (Additional file 6). The heterogeneity was not significantly decreased after the subgroup analysis. The TK at the last follow-up was significantly greater in the MISS group for patients with > 10 fusion levels (WMD, 4.24; 95% CI 1.11 to 7.36, *P* = 0.008). The EBL was significantly lower in the MISS group for patients with > 10 fusion levels (WMD, -213.09; 95% CI -296.40 to -129.79, *P* < 0.001) and ≤ 10 fusion levels (WMD, -275.72; 95% CI -333.54 to -217.89, *P* < 0.001). The ORT was significantly longer in the MISS group for patients with > 10 fusion levels (WMD, 100.33; 95% CI 22.14 to 178.53, *P* = 0.012) and ≤ 10 fusion levels (WMD, 75.82; 95% CI 53.86 to 97.79, *P* < 0.001).

## Discussion

Recently, several studies have demonstrated that AIS could be successfully corrected using minimally invasive techniques [11, 14, 15, 19, 32]. Unlike the percutaneous technique and lateral interbody fusion commonly applied in adult degenerative spinal deformity, MISS for AIS used one to three small skin incisions from a posterior approach, which allowed surgeons to perform facetectomy, instrumentation, and fusion [15, 33]. Compared to standard PSF, dissection of soft tissue and the spinal posterior structure was less common, and the cosmetic concerns of adolescent patients could be relieved [19, 32]. However, as a novel technique, the effectiveness of MISS for the management of AIS still needs to be clarified. In this study, we found that MISS was associated with adequate deformity correction, better restoration of TK, less EBL, lower blood transfusion rate, shorter LOS, and less pain than PSF.

### Correction in the coronal and sagittal planes

Due to the limited available instrumentation for MISS, the application of compression, distraction, and in situ contouring are still challenges for this technique [31]. However, MISS yielded correction of the main curve Cobb angle, LL, CB, and SVA that was comparable to standard PSF in this meta-analysis. The better restoration of TK in the MISS group, especially for patients with > 10 fusion levels, was an unexpected finding, although the difference was minor, and whether it had any clinical significance remained debatable. Recently, Sarwahi et al. reported that MISS was associated with significantly greater restoration of TK and a lower postoperative hypokyphosis rate than standard PSF [21]. One plausible explanation was that the preservation of paraspinal muscles and ligaments in the MISS procedure played an important role in maintaining sagittal alignment after surgery [35]. Overall, MISS was adequate to achieve the goal of deformity correction in both the coronal and sagittal planes.

### Estimated blood loss

Minimizing blood loss is of paramount importance for the surgical management of spinal deformity, as the use of allogenic blood transfusion is a risk factor for the development of surgical site infection after spinal fusion [36]. The impact of bleeding-related and transfusion-related morbidity is even more pronounced in pediatric and adolescent populations. The smaller body size of these patients could accelerate the proportional loss of blood volume during deformity correction surgery for AIS [37]. In a cohort of 188 patients with AIS, Ialenti et al. reported a mean EBL of 907 ± 775 mL with standard PSF [34]. In another large cohort of 43,983 patients who underwent PSF for AIS, Yoshihara and Yoneoka reported a blood transfusion rate of 30.4% [38]. Chiu et al. showed that screw insertion and derotation maneuvers were the main contributors to EBL during standard PSF [39]. These two steps were not modified during the MISS procedure; nonetheless, the EBL and the corresponding need for blood transfusion were significantly decreased in the current study, with an EBL of 288.25 mL and a blood transfusion rate of 8.0%. We believe that the decreased EBL in the MISS group was attributable to the smaller incisions, reduced tissue dissection, and smaller area of subperiosteal exposure [14]. The ORT was significantly longer in the MISS group than in the PSF group in this study, which reflected the learning curve when applying the new technique [32]. As the longer ORT could also contribute to the increased EBL, we considered that the EBL in the MISS group might continue to decrease with increasing surgeon experience [34, 40].

### Length of hospital stay, postoperative pain, and SRS-22 score

This meta-analysis found that the LOS was shorter by 1.48 days in the MISS group than in the PSF group. We considered that this positive outcome was related to the reduced dissection of muscle and EBL in the MISS procedure, which enhanced patient recovery and allowed early discharge. Sultan et al. reported that intraoperative blood loss was an independent risk factor for prolonged LOS in AIS patients undergoing PSF because patients with increased EBL were more likely to develop postoperative complications and require additional management [41]. The shorter LOS may also be tied to patients’ postoperative pain and satisfaction [42, 43]. Martin et al. reported that achieving adequate pain control could decrease the opioid requirement and minimize opioid-induced side effects, allowing AIS patients to mobilize early [44]. Additionally, patients with higher satisfaction tend to return home faster [44]. Consistent with previous studies, we found that patients in the MISS group reported significantly less pain, according to the SRS-22 pain score (4.31 vs. 3.80) and the VAS score (5.94 vs. 6.28), which was attributed to the reduced dissection of tissue with this new technique. Although the results were not statistically significant, the SRS-22 self-image/appearance score was also higher in the MISS group than in the PSF group (4.05 vs. 3.95). Better pain management and scar appearance could improve overall patient satisfaction, which was reflected by the higher overall SRS-22 score in the MISS group (4.22 vs. 4.11) and led to the shorter LOS in this study.

### Complications

The overall complication profile is an essential aspect of the safety evaluation of a novel surgical technique, particularly in the early stage of application. In this study, the MISS yielded a complication spectrum and complication rate similar to those of standard PSF (15.0% vs. 18.0%), suggesting that this new procedure would not cause additional harm and was a safe alternative to conventional open surgery. The most commonly reported complication in both groups was surgical site infection. In this study, the surgical site infection rate was 2.0% in the MISS group, similar to the PSF group. In a previous study of 540 AIS patients who underwent standard PSF, 2.8% of cases developed postoperative surgical site infection, which was consistent with our results [45]. Additionally, surgical site infection is associated with ORT [14]. We believe that the decreased ORT of the MISS procedure with increasing surgeon experience would lead to a decrease in the surgical site infection rate.

### Concerns regarding the MISS procedure

Despite MISS’s advantages of better restoration of TK, less EBL, fewer transfusions, shorter LOS, and less postoperative pain, several important concerns must be acknowledged. The ORT was significantly longer with MISS than with PSF (376.40 min vs. 287.60 min). The difference in ORT may be the effect of the steep learning curve, and de Bodman et al. and Zhu et al. reported that the ORT of the MISS procedure could be shortened by nearly 60 min with increased surgeon experience [32, 40]. Nonetheless, even when MISS is performed by a proficient surgeon, the ORT might still be slightly longer than that of standard PSF, and this should be emphasized as a potential limitation of MISS for AIS [33]. Prolonged surgical times keep patients under extended anesthesia, which may have deleterious effects on perioperative morbidity [18].

Concerns have been raised that more fluoroscopic imaging may be needed during the MISS procedure, which could increase the radiation exposure of both patients and surgeons [32, 33]. However, Si et al. reported that a satisfactory screw placement rate of 93.6% could be obtained using the freehand technique, and no significant difference in radiation exposure was found between the MISS group and the PSF group [19]. Additionally, Zhu et al. achieved a satisfactory screw placement rate of 93.8% in the MISS group through O-arm navigation, and patients and surgeons could be exposed to less radiation from an O-arm than with traditional fluoroscopy [32].

Appropriate patient selection is important for a new technique, although little is known about the indications of the MISS procedure. To date, most studies have focused on moderate (< 80°) and flexible (> 50%) AIS [14, 15, 19, 20, 31, 32]. Whether the indication can be extended to patients with more severe and rigid curves remains unclear.

### Limitations

This study had several limitations. First, the heterogeneity of the included patients should be acknowledged, as they had different curve types and fusion levels. Two studies focused on Lenke types 1–4, two studies focused on Lenke type 5, the other three studies included mixed curve types (Lenke type 1–6), and the fusion levels in these studies ranged from 5 to 12. Heterogeneity may have impacted meaningful pooling. Although we performed subgroup analysis, heterogeneity was still present. Other sources of heterogeneity may have been differences in surgeons, management protocols of spinal centers, and study designs. Second, it is important to address the inherent potential for bias underlying the use of retrospective studies of MISS versus PSF. It is clear that in the absence of randomization, patient selection for MISS or PSF is likely to be a key source of bias in all of the included studies. The patients who are most appropriate for MISS are likely to be selected to undergo MISS, and vice versa. Third, because MISS is a new technique, the results reported by the included studies reflected the early experience of surgeons. Regarding the learning curve of the MISS, de Bodman et al. indicated that the outcomes could be improved after the first 25 cases [40]. However, five of the included studies reported data for their first 25 cases [15, 20, 31–33]. Therefore, the results of this study may be limited when surgeons gain more experience.

## Conclusions

Despite inherent technical challenges, MISS is a feasible and effective alternative to standard PSF for AIS patients with moderate and flexible curves. MISS was associated with adequate deformity correction, better restoration of sagittal alignment, less EBL, fewer transfusions, shorter LOS, and better pain management than PSF. Further research is required to determine the detailed indications for the MISS procedure.

## Supplementary Information

**Additional file 1**. Galbraith plot of the correction rate.

**Additional file 2**. Influence analysis of the correction rate.

**Additional file 3**. Galbraith plot of the estimated blood loss.

**Additional file 4**. Influence analysis of the estimated blood loss.

**Additional file 5**. Subgroup analysis by curve type.

**Additional file 6**. Subgroup analysis by fusion levels

**Additional file 7**. PRISMA 2009 Checklist.

## Data Availability

The datasets used and analyzed in the current study can be obtained from the corresponding author on reasonable request.

## Abbreviations

AIS: Adolescent idiopathic scoliosis
PSF: Posterior spinal fusion
MIS: Minimally invasive surgery
MISS: Minimally invasive scoliosis surgery
EBL: Estimated blood loss
ORT: Operative time
LOS: Length of hospital stay
TK: Thoracic kyphosis
LL: Lumbar lordosis
CB: Coronal balance
SVA: Sagittal vertical axis
VAS: Visual analog scale
SRS-22: Scoliosis Research Society-22
WMD: Weighted mean difference
RR: Risk ratio
PRISMA: Preferred Reporting Items for Systematic Reviews and Meta-Analyses
