# Supplementary figures and images for: Posterior minimally invasive scoliosis surgery versus the standard posterior approach for the management of adolescent idiopathic scoliosis: an updated meta-analysis
Source: J Orthop Surg Res. 2022 Jan 29;17:58. doi: 10.1186/s13018-022-02954-4 (PMC8800201; doi:10.1186/s13018-022-02954-4)

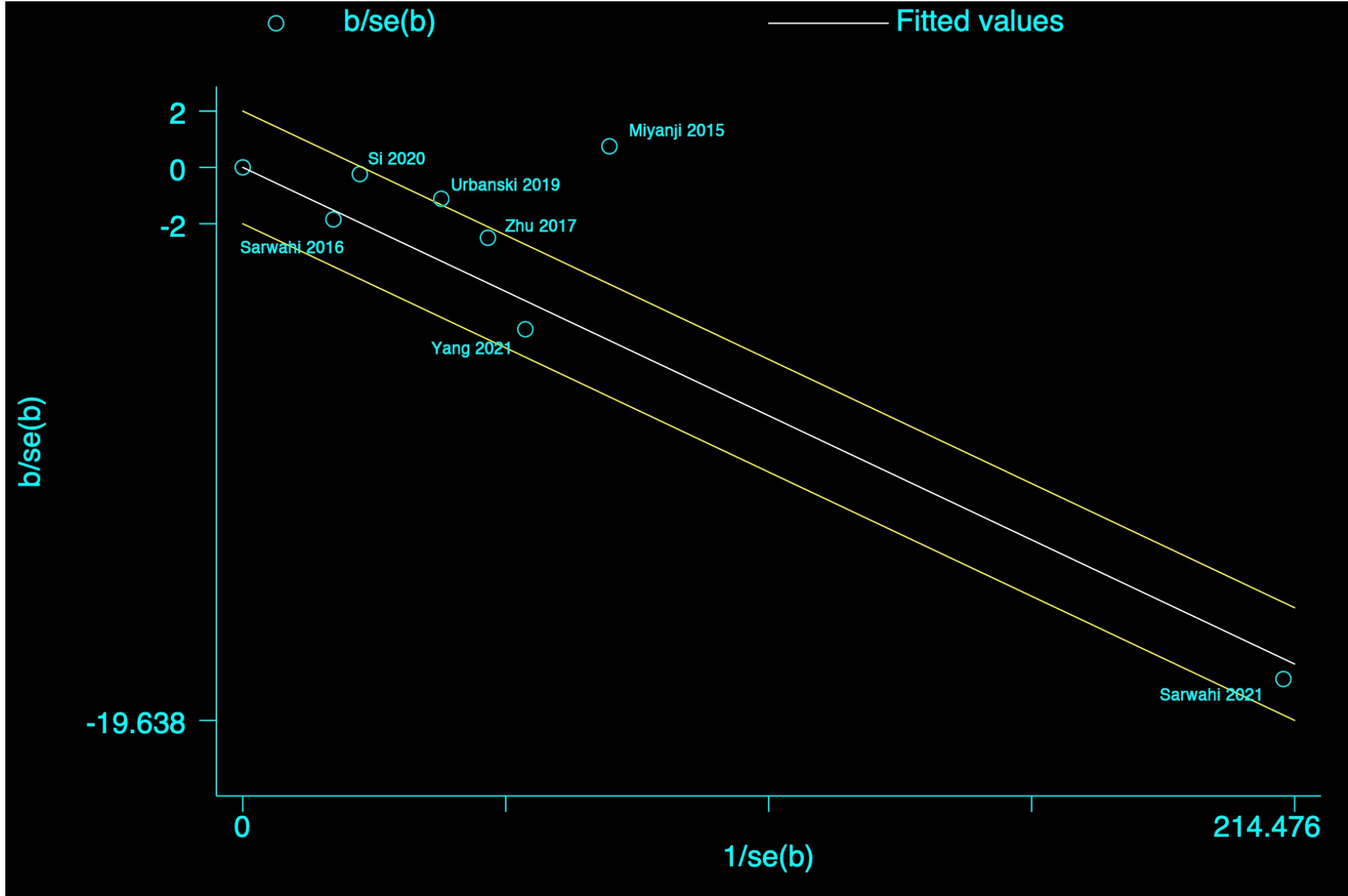

Additional file 1. Galbraith plot of the correction rate

Supplement: Supplementary file 1 — Additional file 1. Galbraith plot of the correction rate. [file 13018_2022_2954_MOESM1_ESM.pdf]

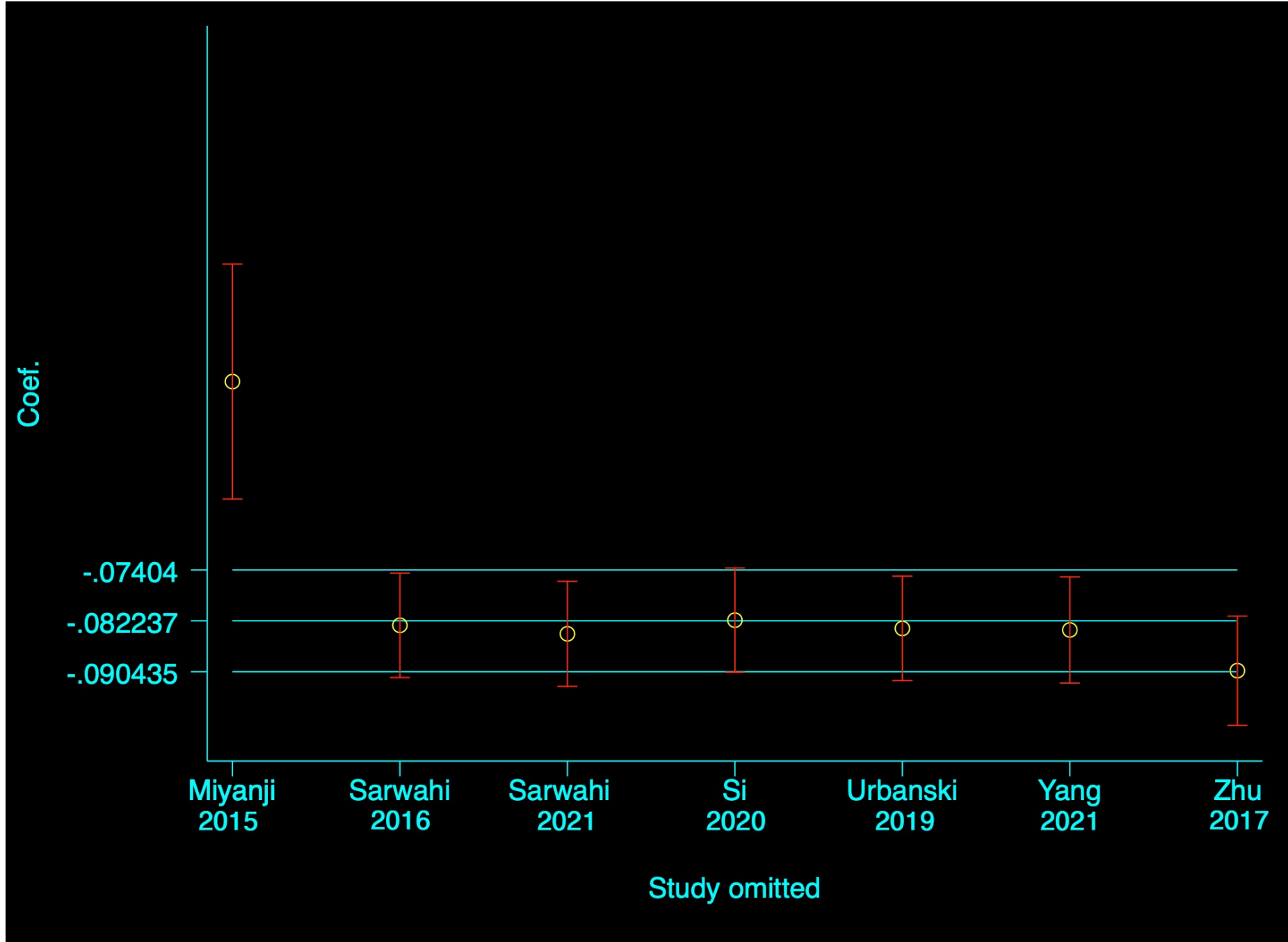

Additional file 2. Influence analysis of the correction rate

Supplement: Supplementary file 2 — Additional file 2. Influence analysis of the correction rate. [file 13018_2022_2954_MOESM2_ESM.pdf]

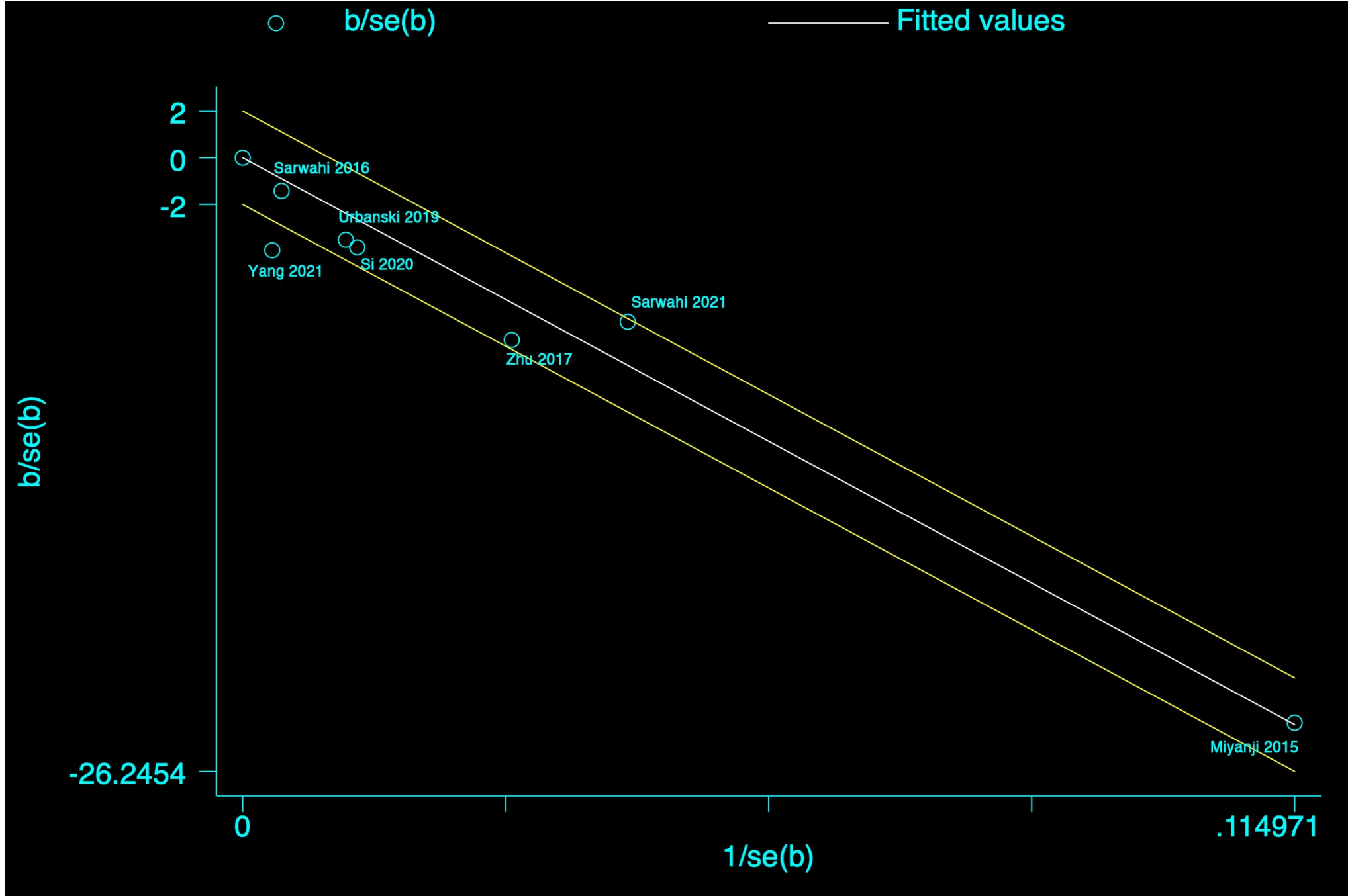

Additional file 3. Galbraith plot of the estimated blood loss

Supplement: Supplementary file 3 — Additional file 3. Galbraith plot of the estimated blood loss. [file 13018_2022_2954_MOESM3_ESM.pdf]

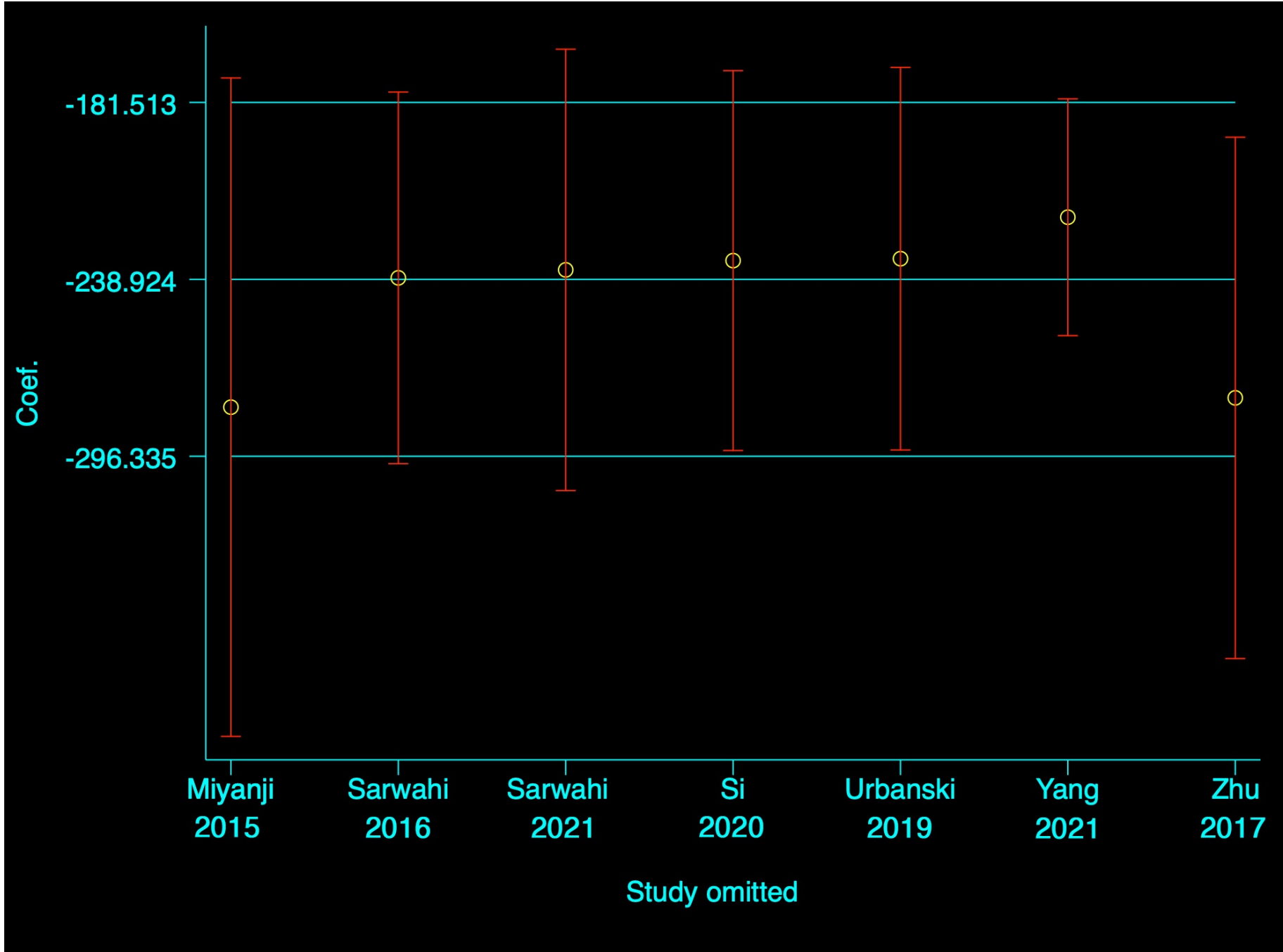

Additional file 4. Influence analysis of the estimated blood loss

Supplement: Supplementary file 4 — Additional file 4. Influence analysis of the estimated blood loss. [file 13018_2022_2954_MOESM4_ESM.pdf]
