# Supplementary material for: Posterior minimally invasive scoliosis surgery versus the standard posterior approach for the management of adolescent idiopathic scoliosis: an updated meta-analysis
Source: J Orthop Surg Res. 2022 Jan 29;17:58. doi: 10.1186/s13018-022-02954-4 (PMC8800201; doi:10.1186/s13018-022-02954-4)
Supplement: Supplementary file 5 — Additional file 5. Subgroup analysis by curve type. [file 13018_2022_2954_MOESM5_ESM.pdf]

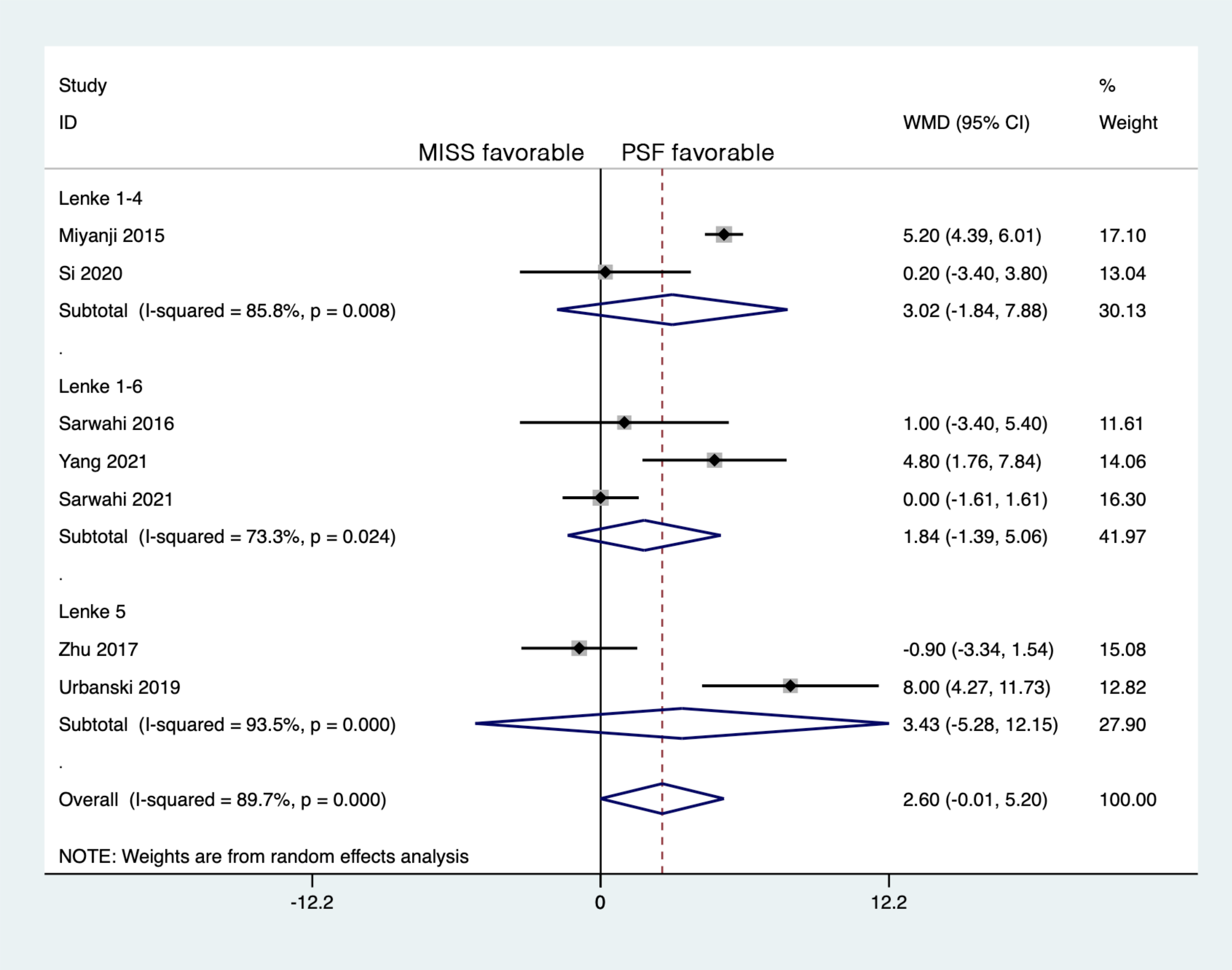

Additional file 5A. Subgroup analysis of the the main curve Cobb angle at the last follow-up according to curve type

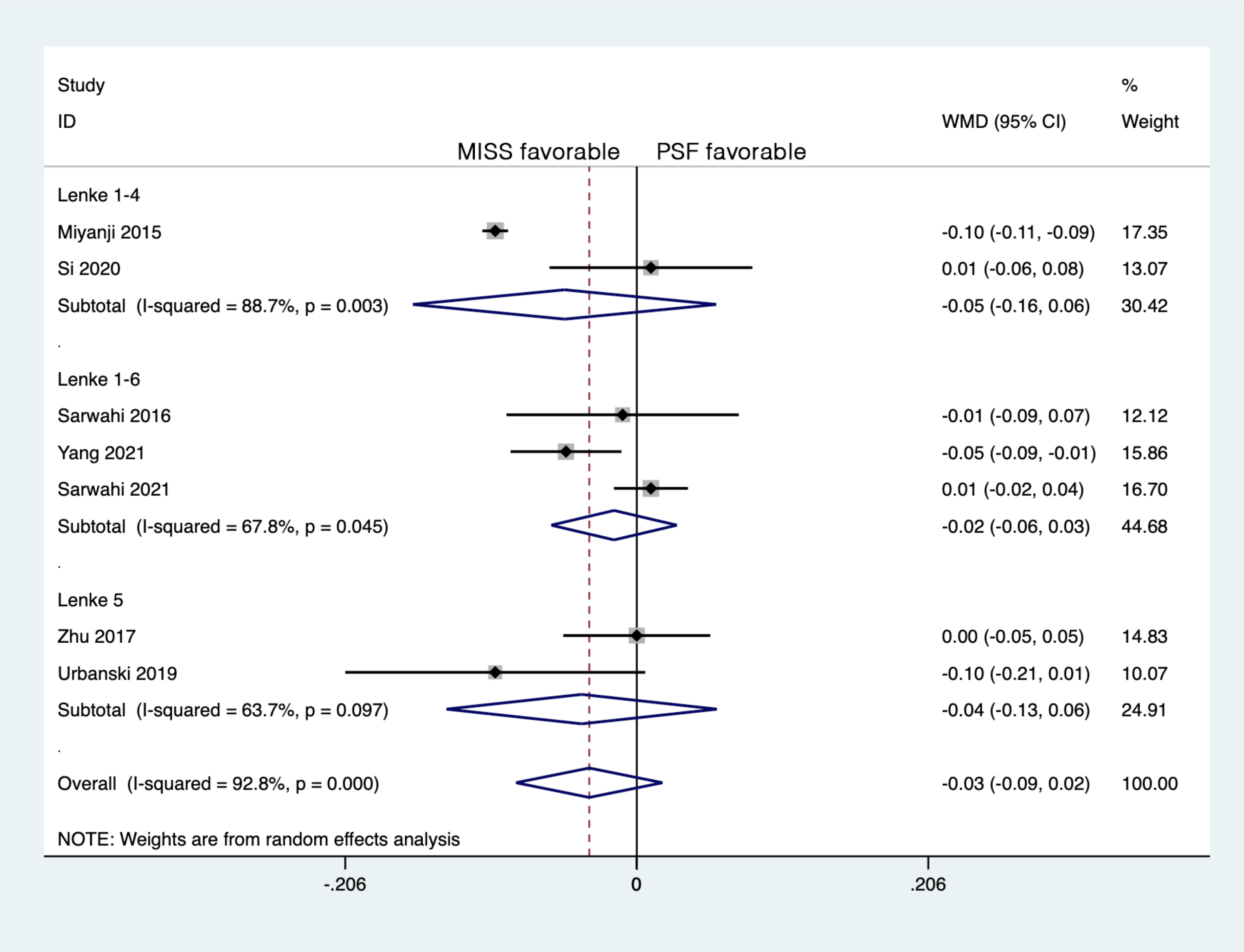

Additional file 5B. Subgroup analysis of the the correction rate according to curve type

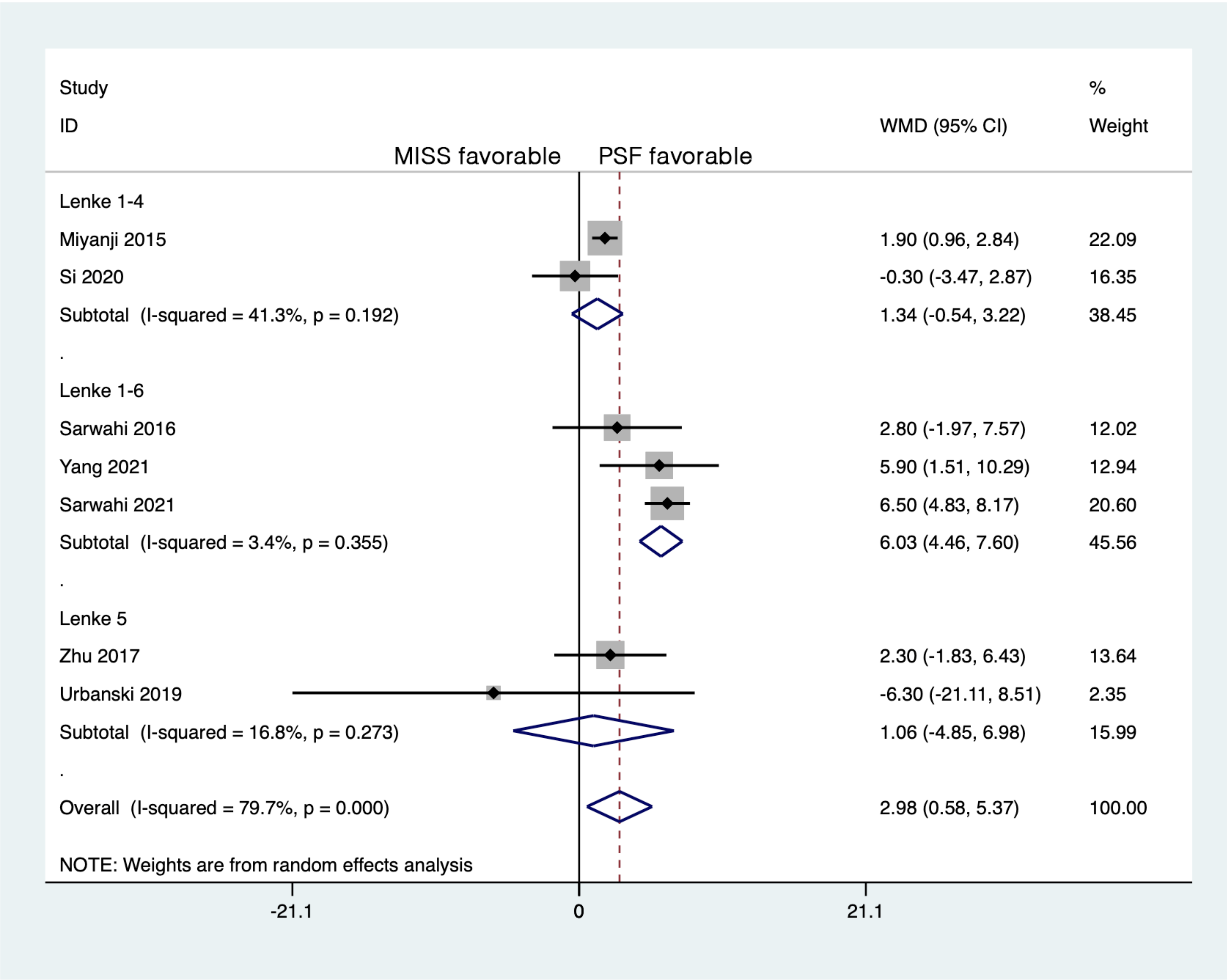

Additional file 5C. Subgroup analysis of the thoracic kyphosis at the last follow-up according to curve type

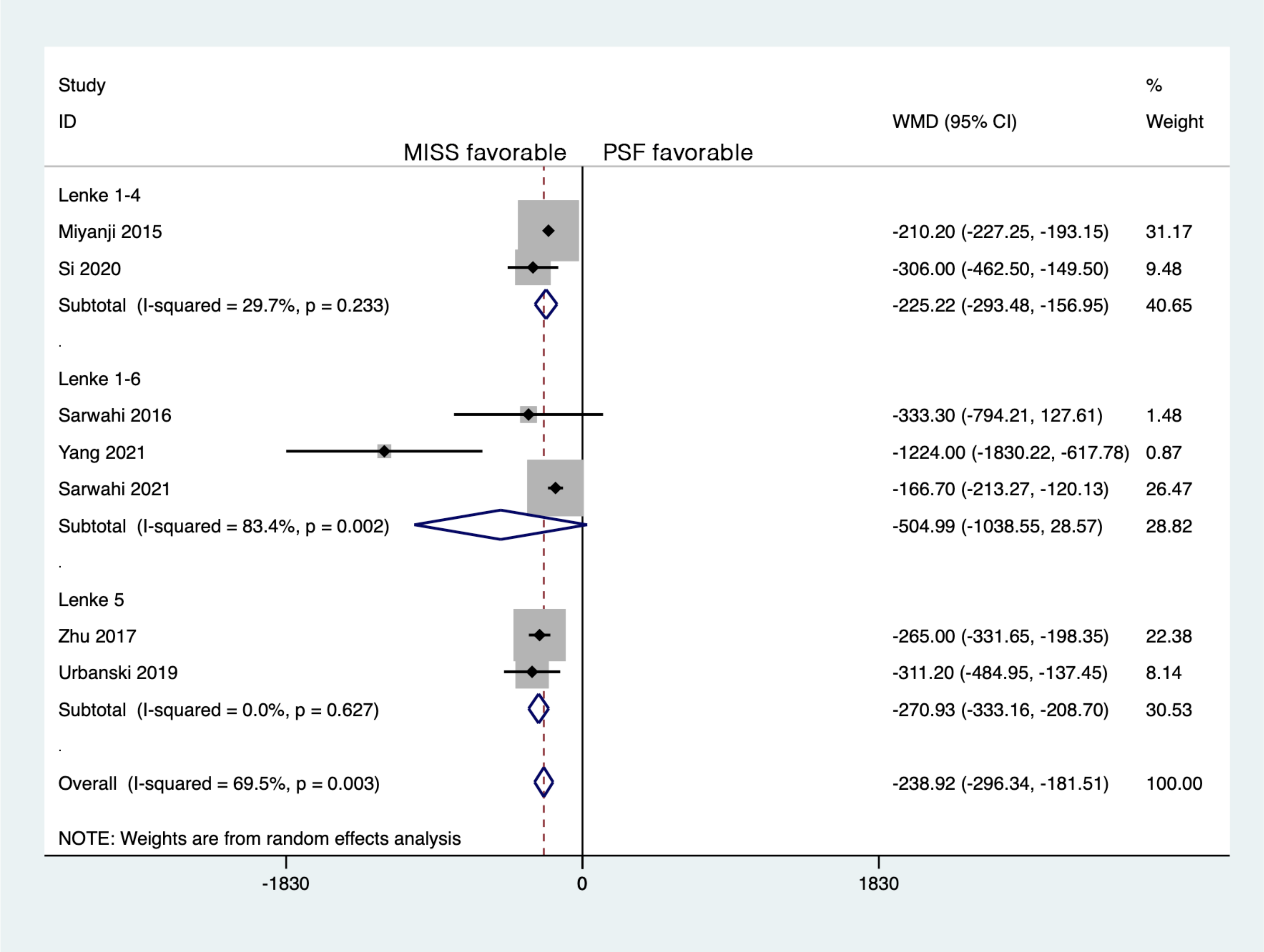

Additional file 5D. Subgroup analysis of the estimated blood loss according to curve type

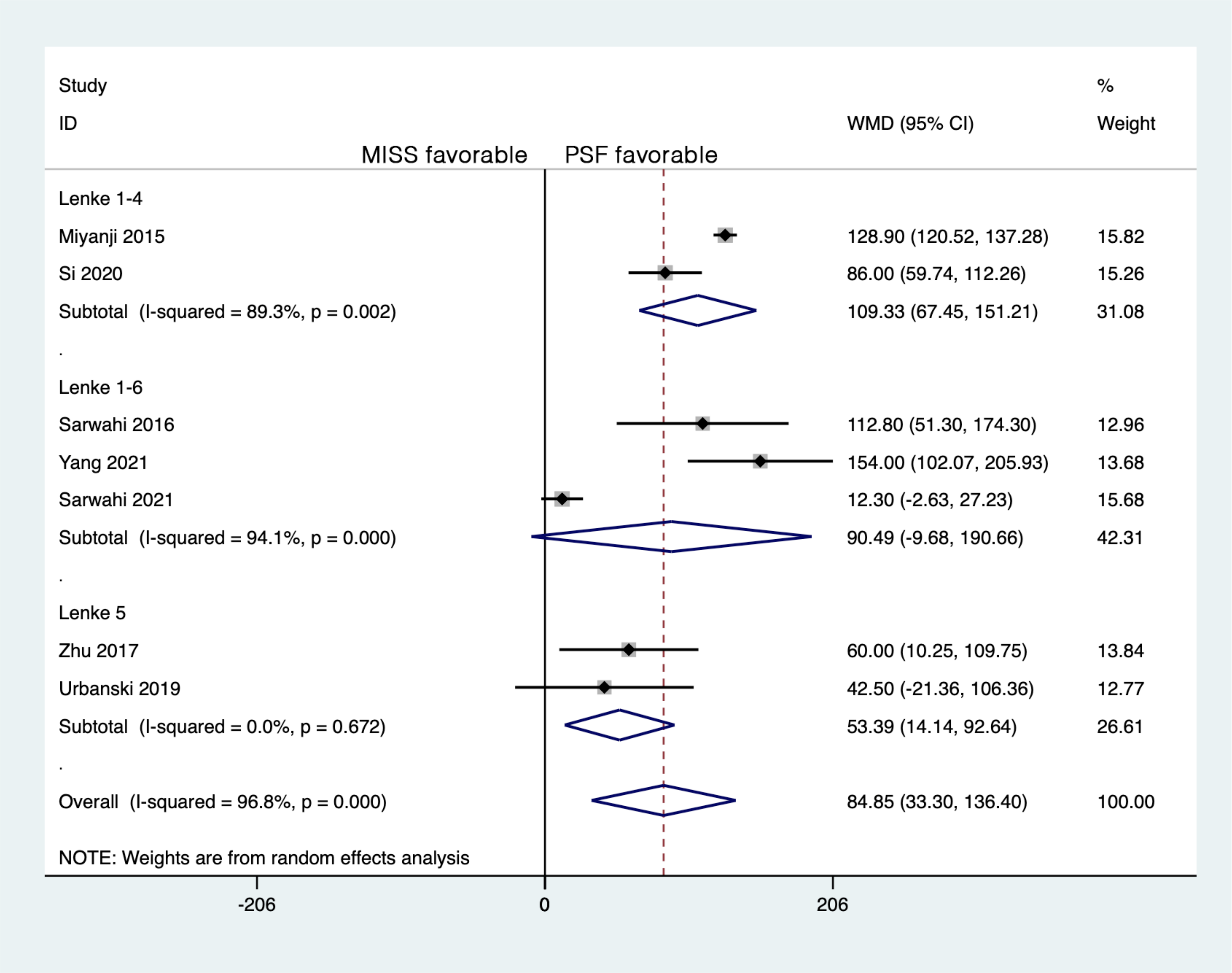

Additional file 5E. Subgroup analysis of the operative time according to curve type
