# Supplementary material for: Posterior minimally invasive scoliosis surgery versus the standard posterior approach for the management of adolescent idiopathic scoliosis: an updated meta-analysis
Source: J Orthop Surg Res. 2022 Jan 29;17:58. doi: 10.1186/s13018-022-02954-4 (PMC8800201; doi:10.1186/s13018-022-02954-4)
Supplement: Supplementary file 6 — Additional file 6. Subgroup analysis by fusion levels [file 13018_2022_2954_MOESM6_ESM.pdf]

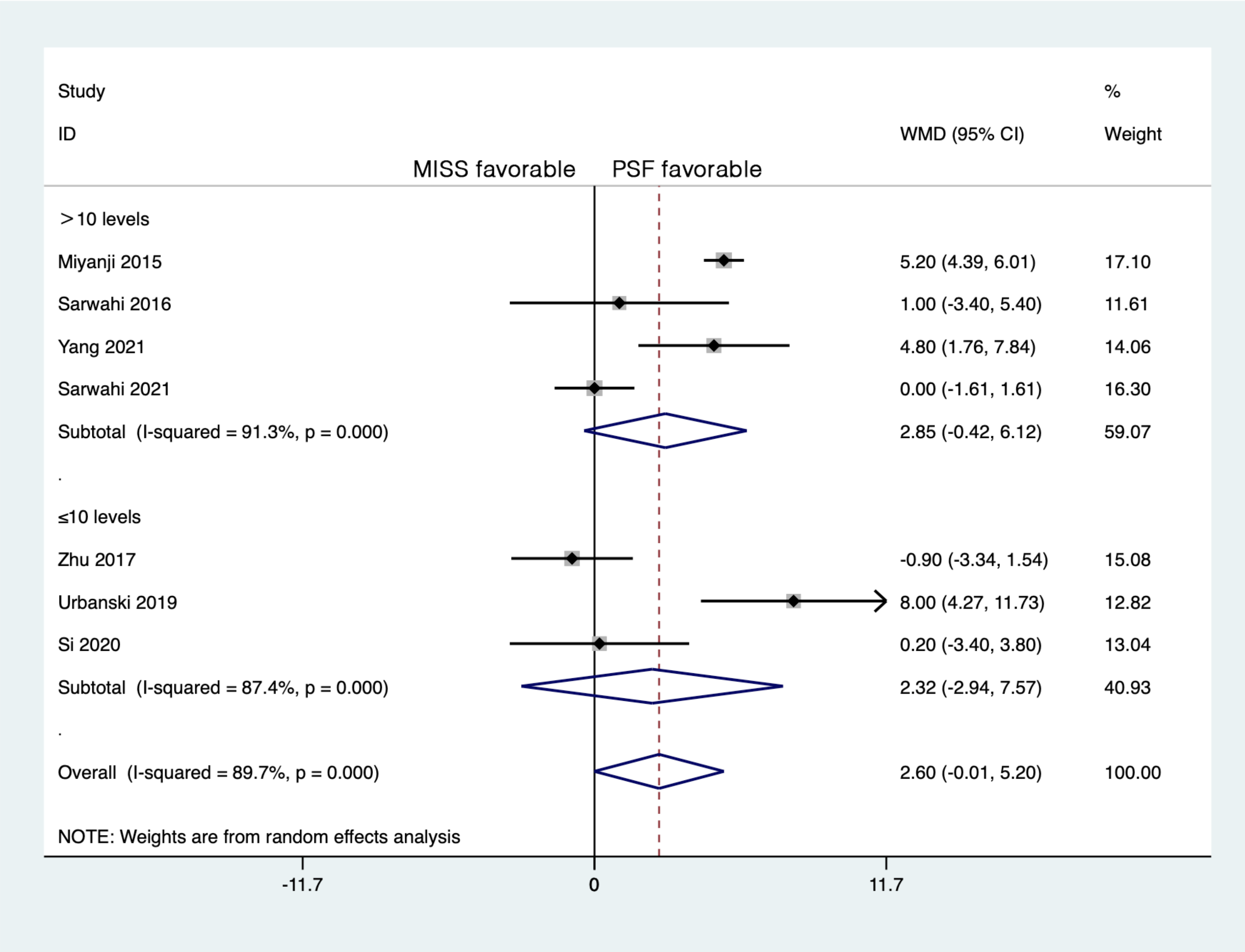

Additional file 6A. Subgroup analysis of the the main curve Cobb angle at the last follow-up according to fusion levels

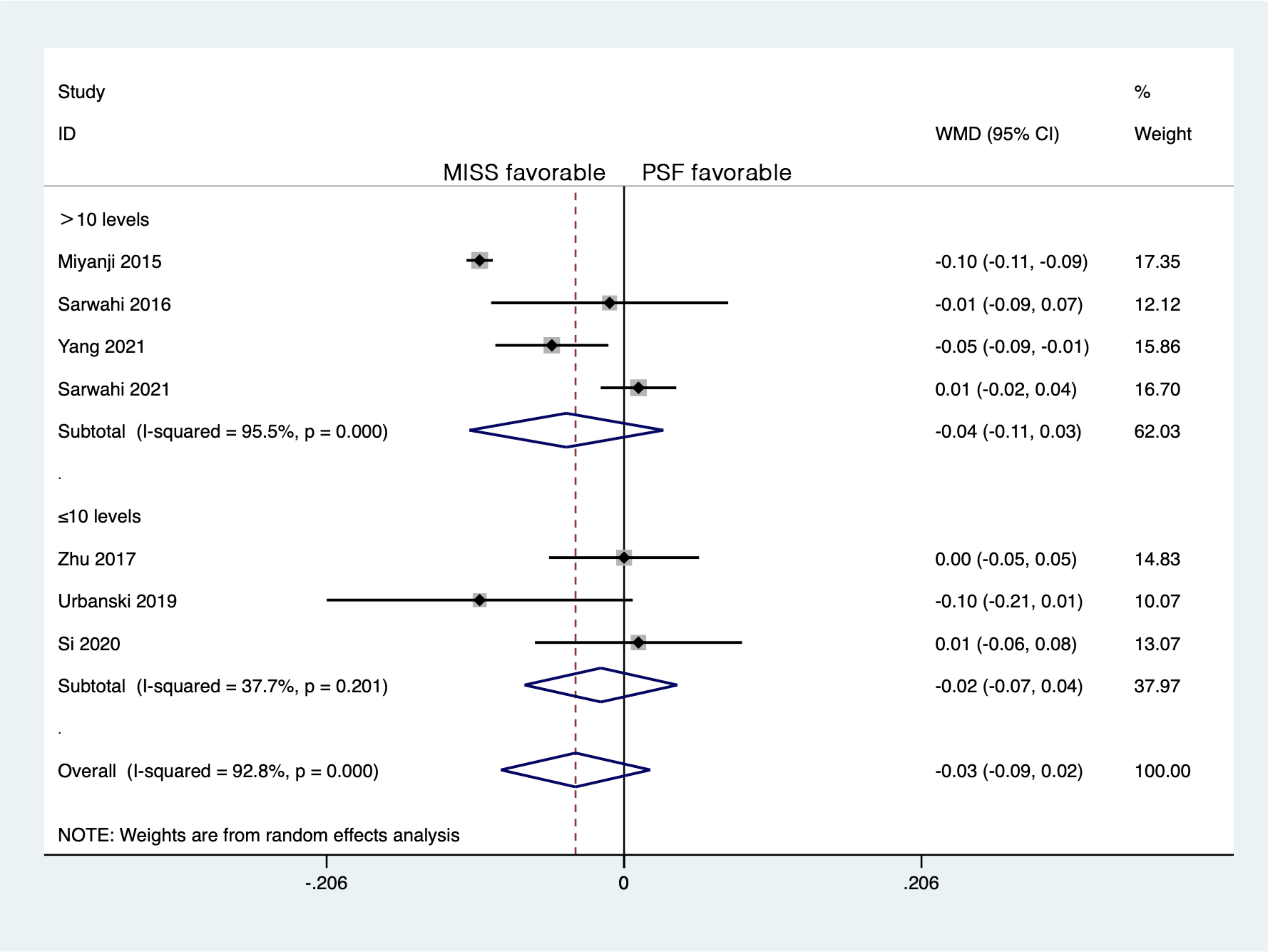

Additional file 6B. Subgroup analysis of the the correction rate according to fusion levels

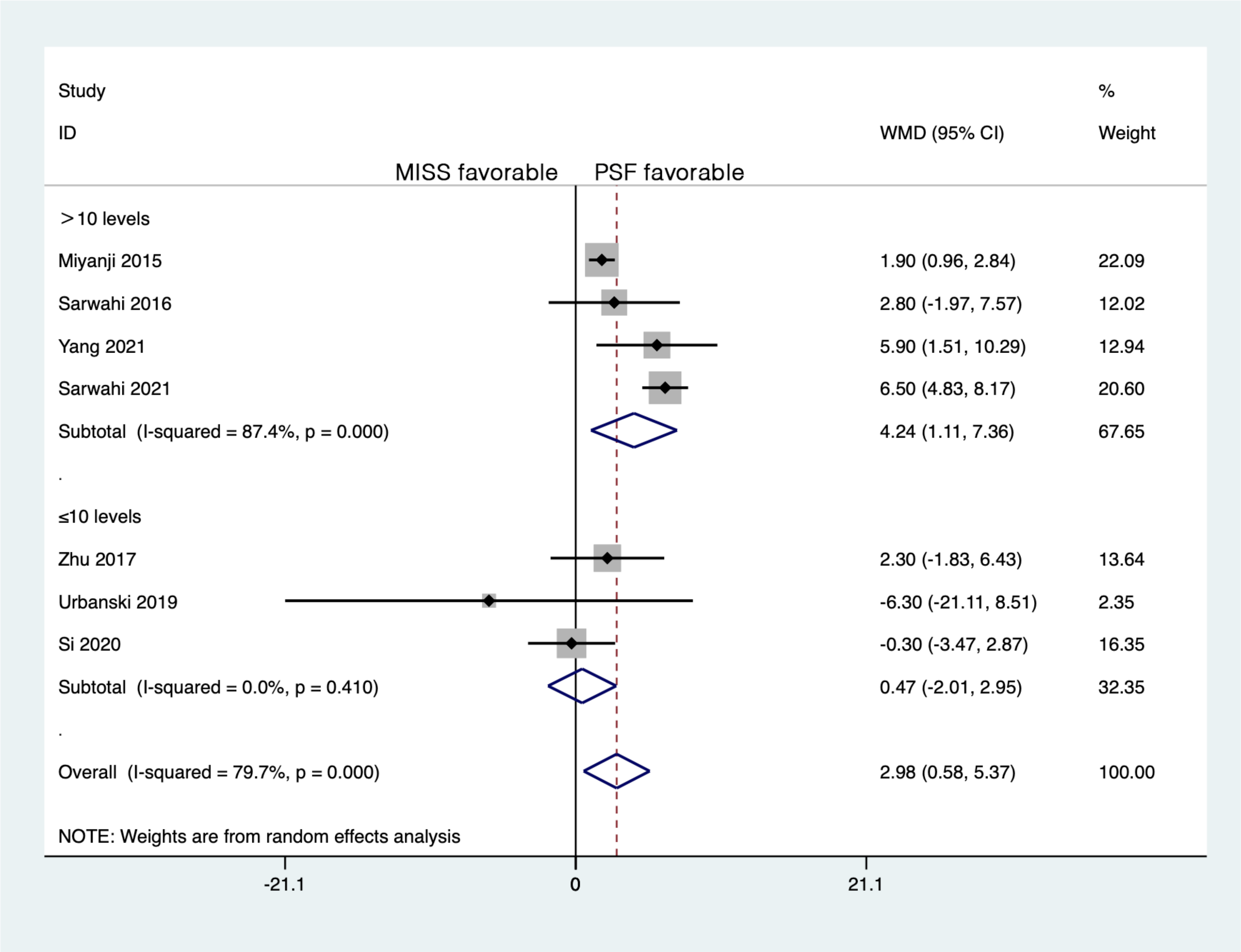

Additional file 6C. Subgroup analysis of the thoracic kyphosis at the last follow-up according to fusion levels

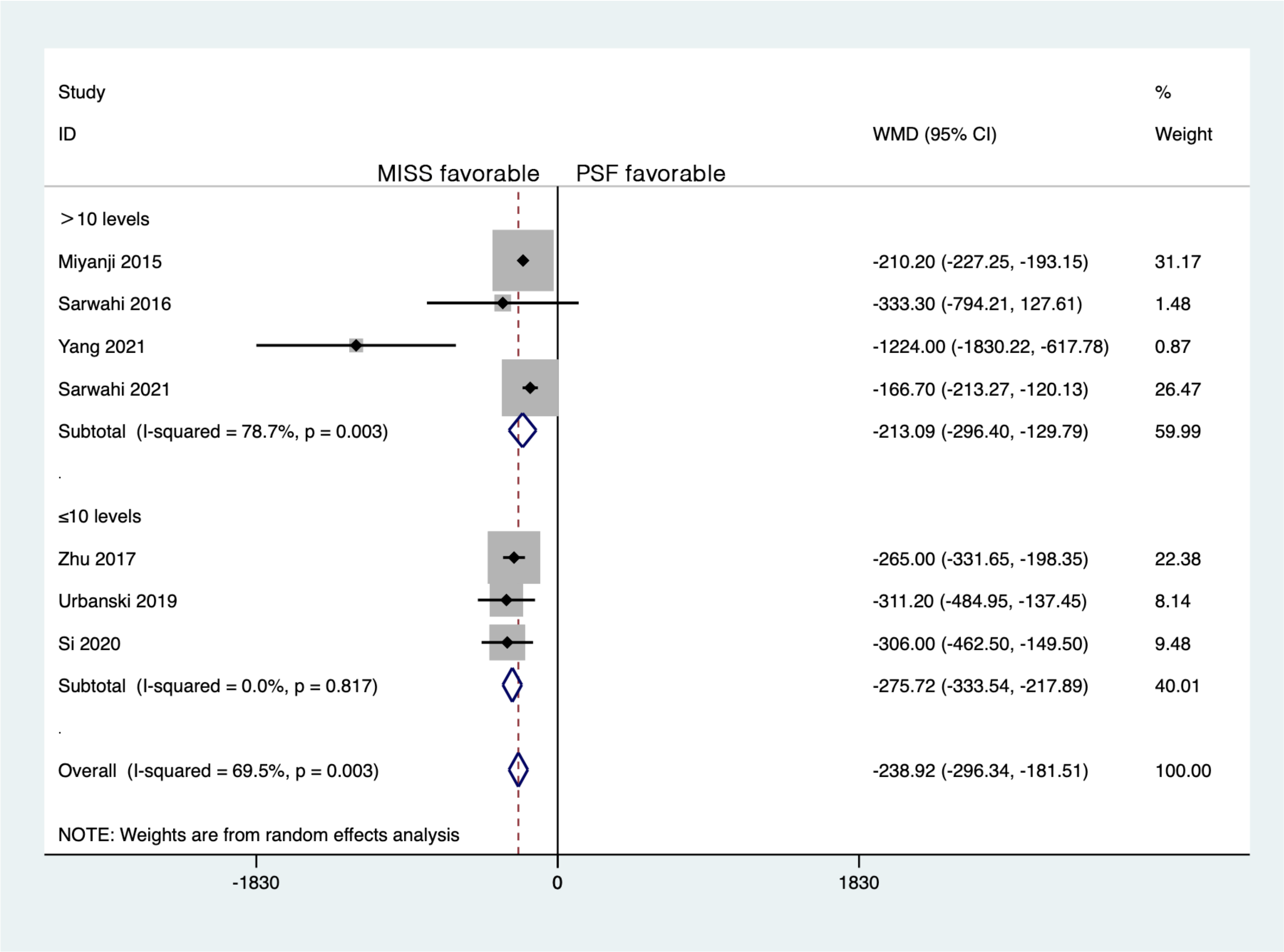

Additional file 6D. Subgroup analysis of the estimated blood loss according to fusion levels

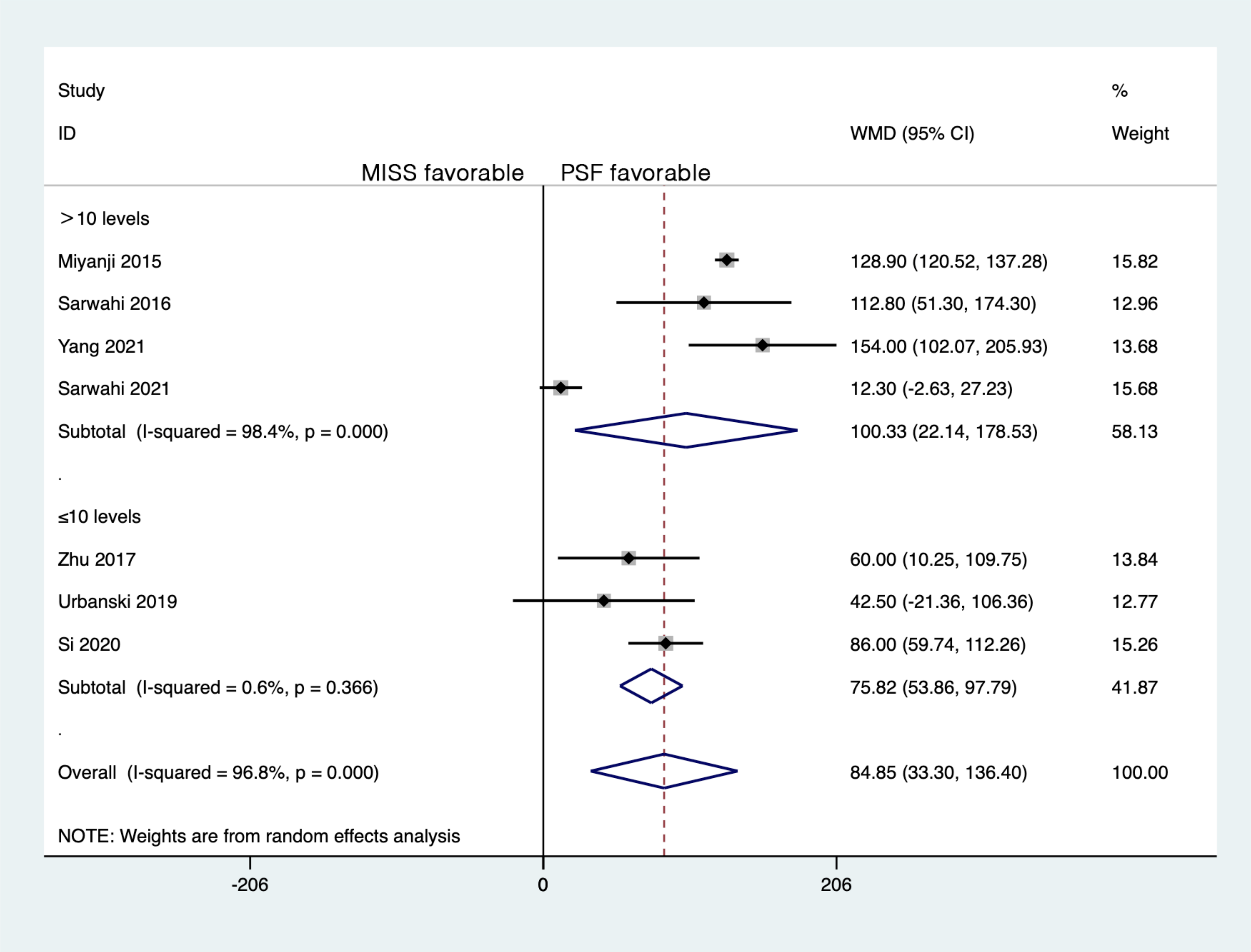

Additional file 6E. Subgroup analysis of the operative time according to fusion levels
